# Supplementary material for: Escherichia coli phylogeny drives co-amoxiclav resistance through variable expression of TEM-1 beta-lactamase
Source: Nat Commun. 2025 Sep 30;16:8669. doi: 10.1038/s41467-025-63714-6 (PMC12484619; doi:10.1038/s41467-025-63714-6)
Supplement: Supplementary file 1 — Supplementary Information [file 41467_2025_63714_MOESM1_ESM.pdf]

Supplementary Figures

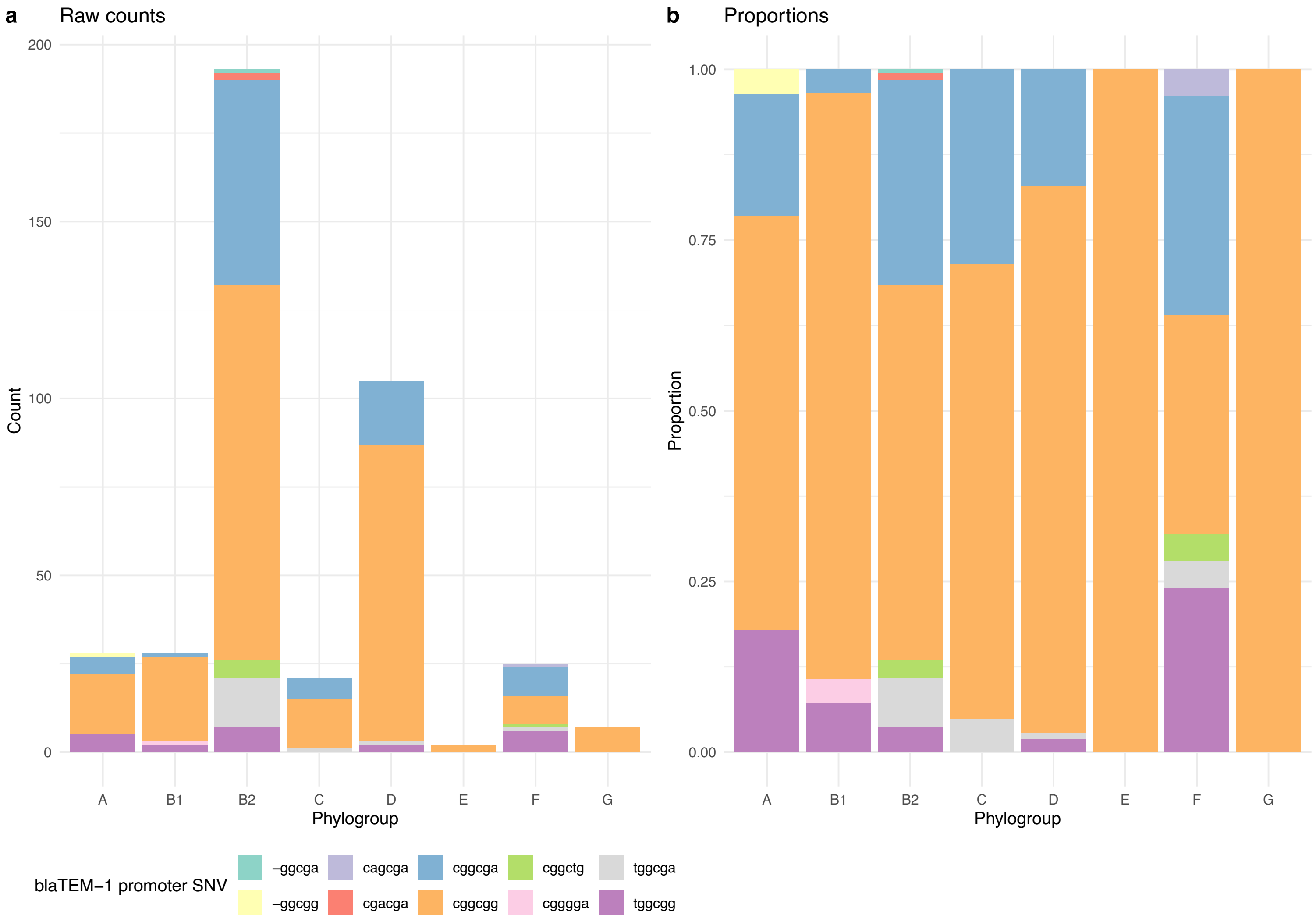

Figure S1. *bla*TEM-1 promoter distribution (*n*=409 promoters). Joint distribution of isolate phylogroup and linked *bla*TEM-1 promoter SNV. Panel (a) shows raw counts and panel (b) shows proportions.

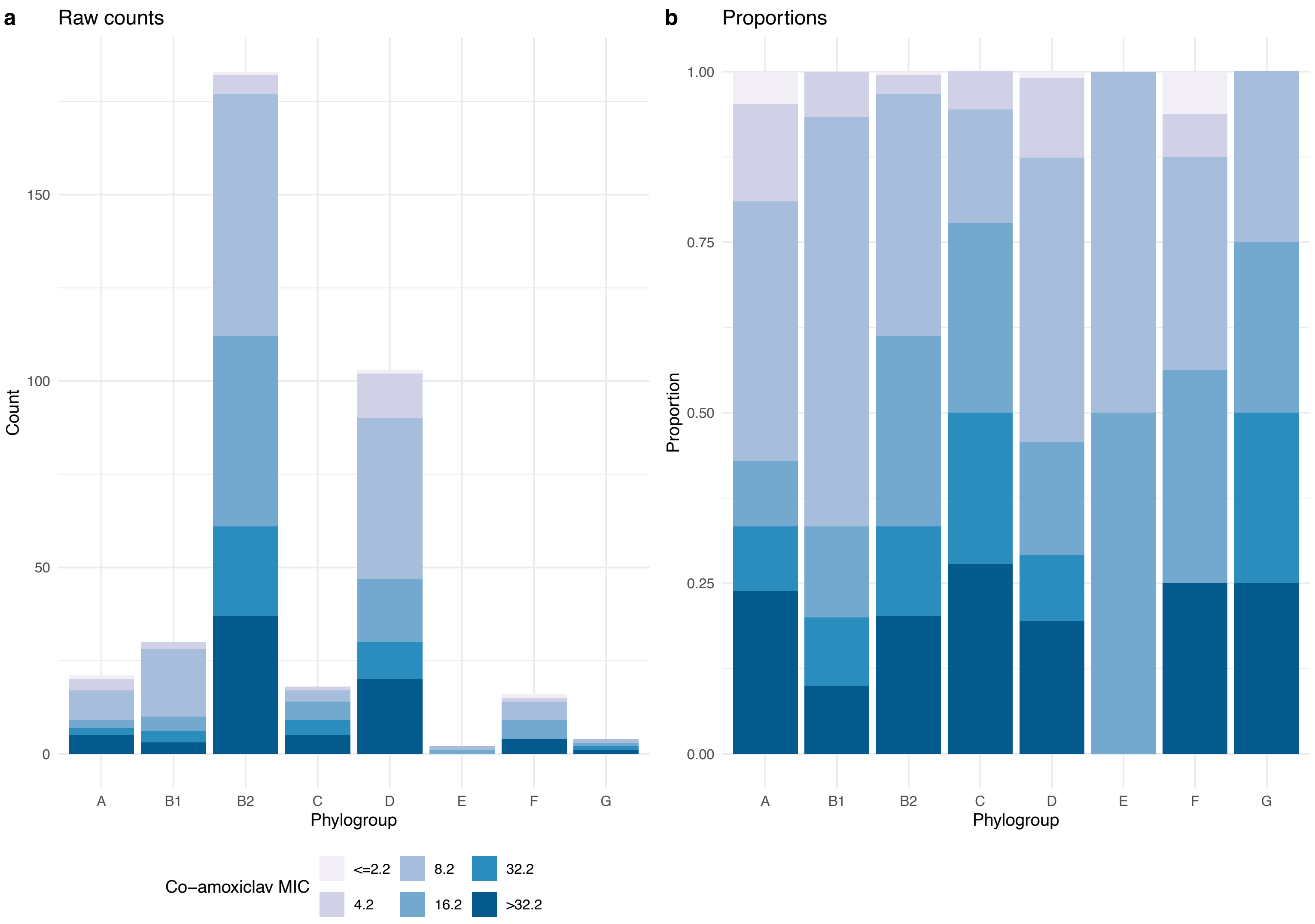

Figure S2. Isolate MIC distribution (*n*=377 isolates). Joint distribution of isolate phylogroup and MIC. Panel (a) shows raw counts and panel (b) shows proportions.

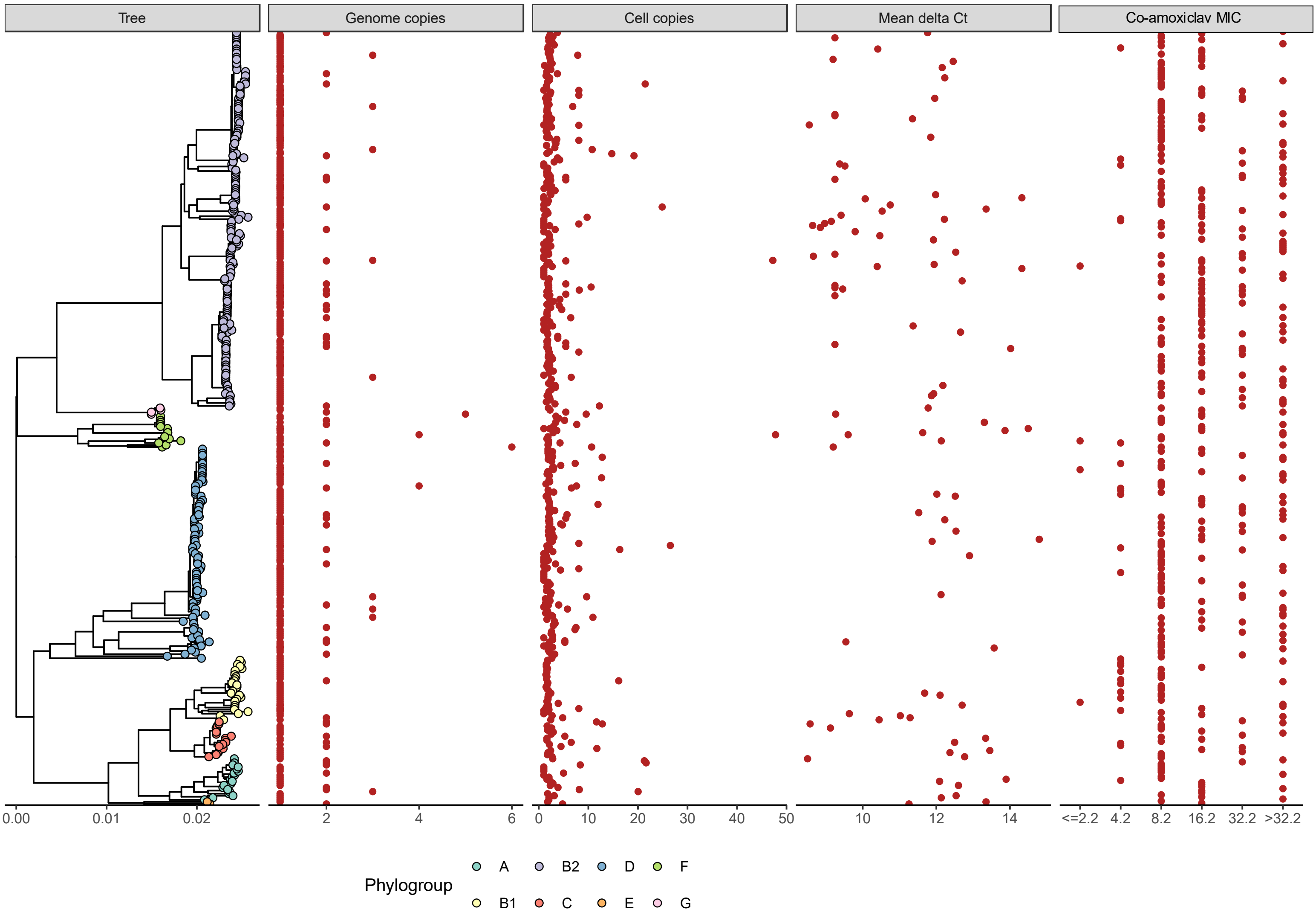

Figure S3. Genotype and phenotype data distribution (*n*=377 isolates). "Tree" panel shows core-gene chromosomal phylogeny with tip coloured by phylogroup. "Genome copies" panel shows bla_{TEM-1} genome copy number. "Cell copies" panel shows bla_{TEM-1} cell copy number. "Mean delta Ct" panel shows bla_{TEM-1} expression qPCR data. "Co-amoxiclav MIC" panel shows isolate MIC.

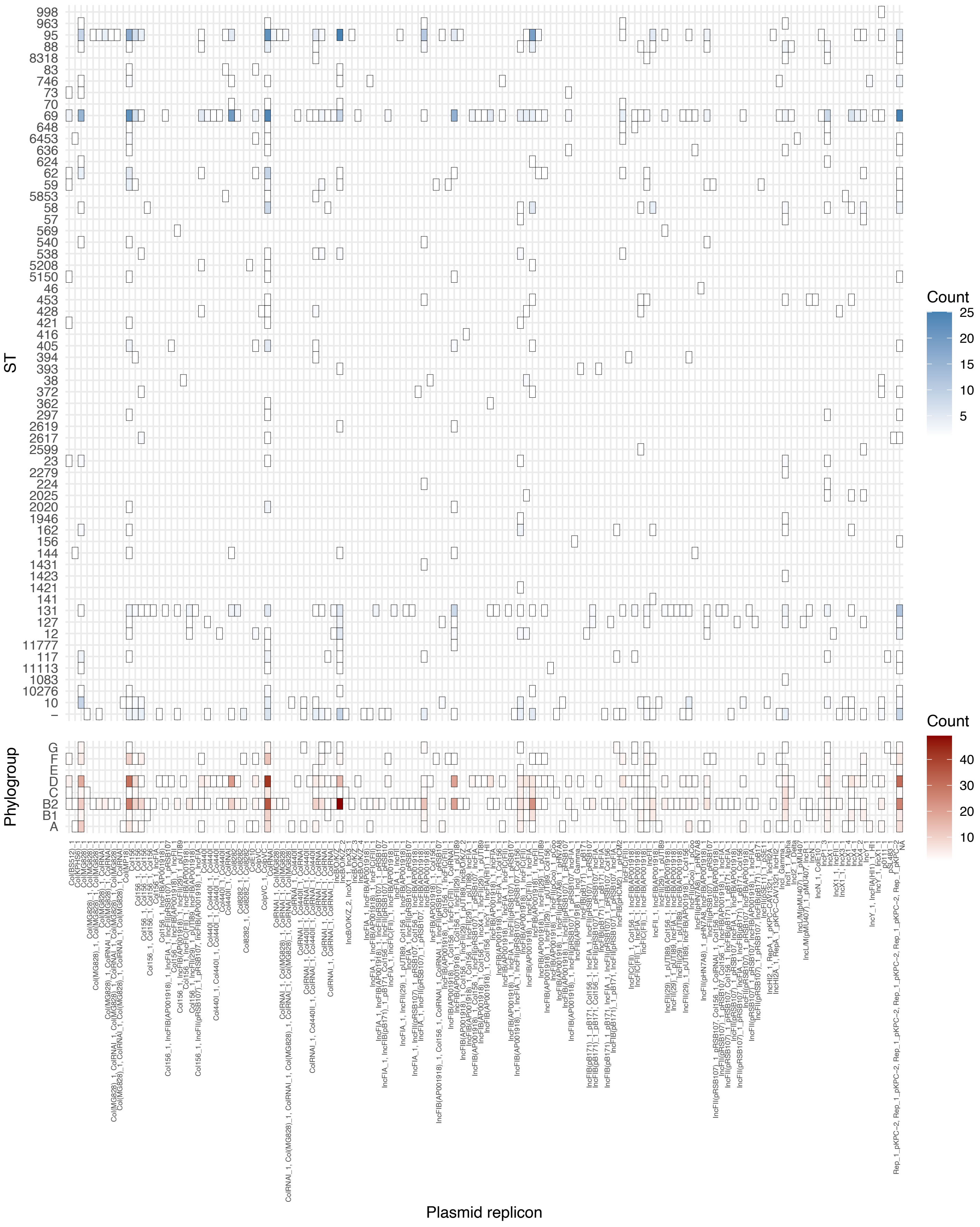

Figure S4. Plasmid replicon type distribution (*n*=1,036 plasmids). Top panel counts PlasmidFinder replicon types by chromosome mlst. Bottom panel counts PlasmidFinder replicon types by chromosome EzClermont phylogroup.

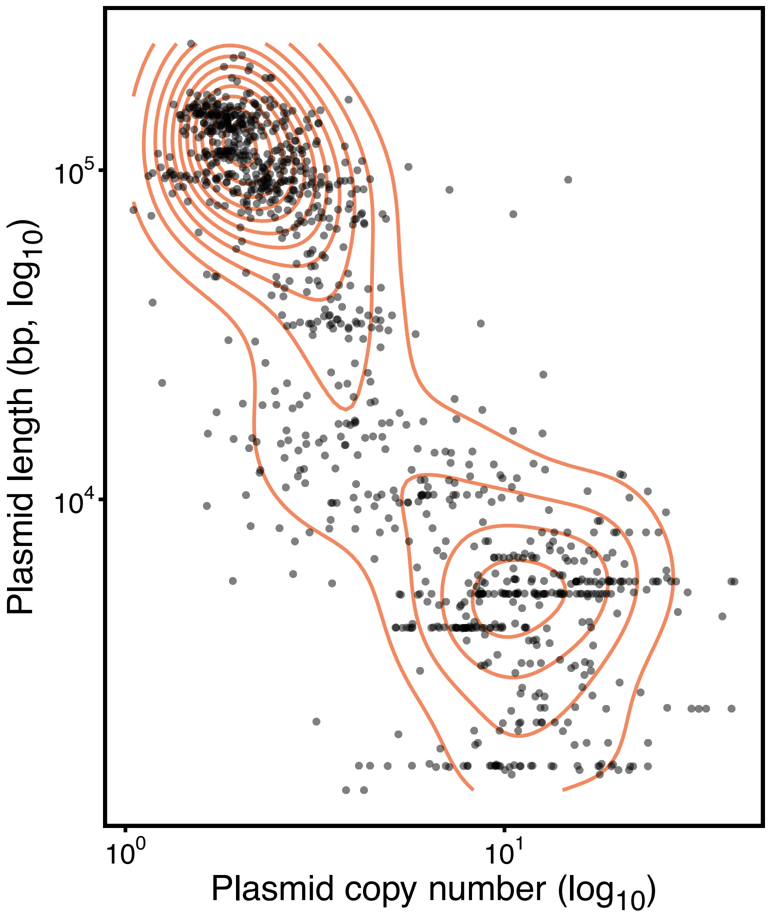

Figure S5. Plasmid copy number distribution (*n*=1,030 plasmids). Plasmid copy number distribution (log_{10}) against plasmid length (bp, log_{10}).

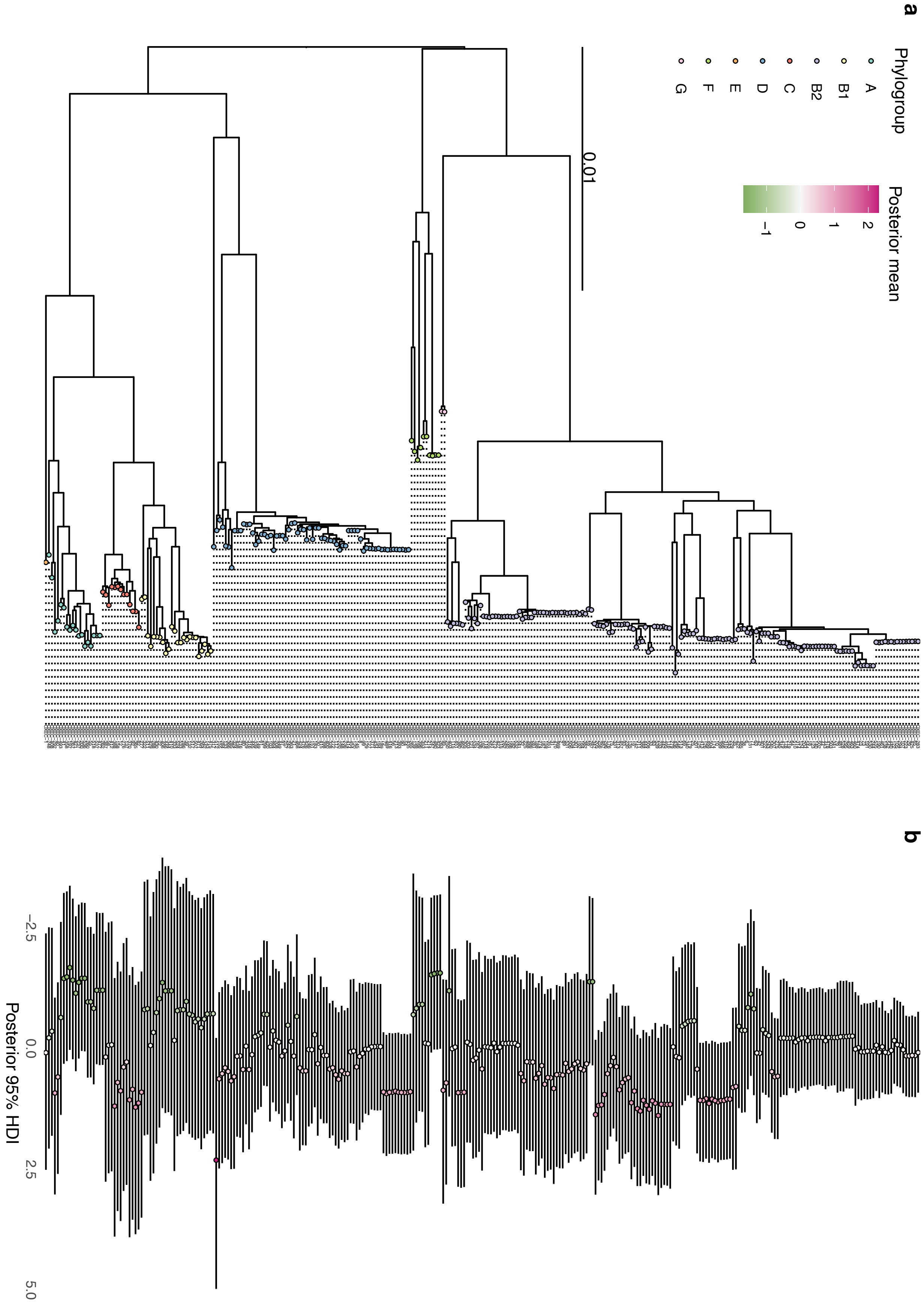

Figure S6. MIC tip effects (*n*=377) (a) A midpoint-rooted core gene phylogeny of *E. coli* chromosomes for all 377 isolates. Tips are coloured by phylogroup. (b) Posterior means (coloured circles) and 95% HPD intervals (horizontal lines) for phylogenetic effect on co-amoxiclav MIC for each tip. Pink indicates above average MIC and green indicates below average MIC.

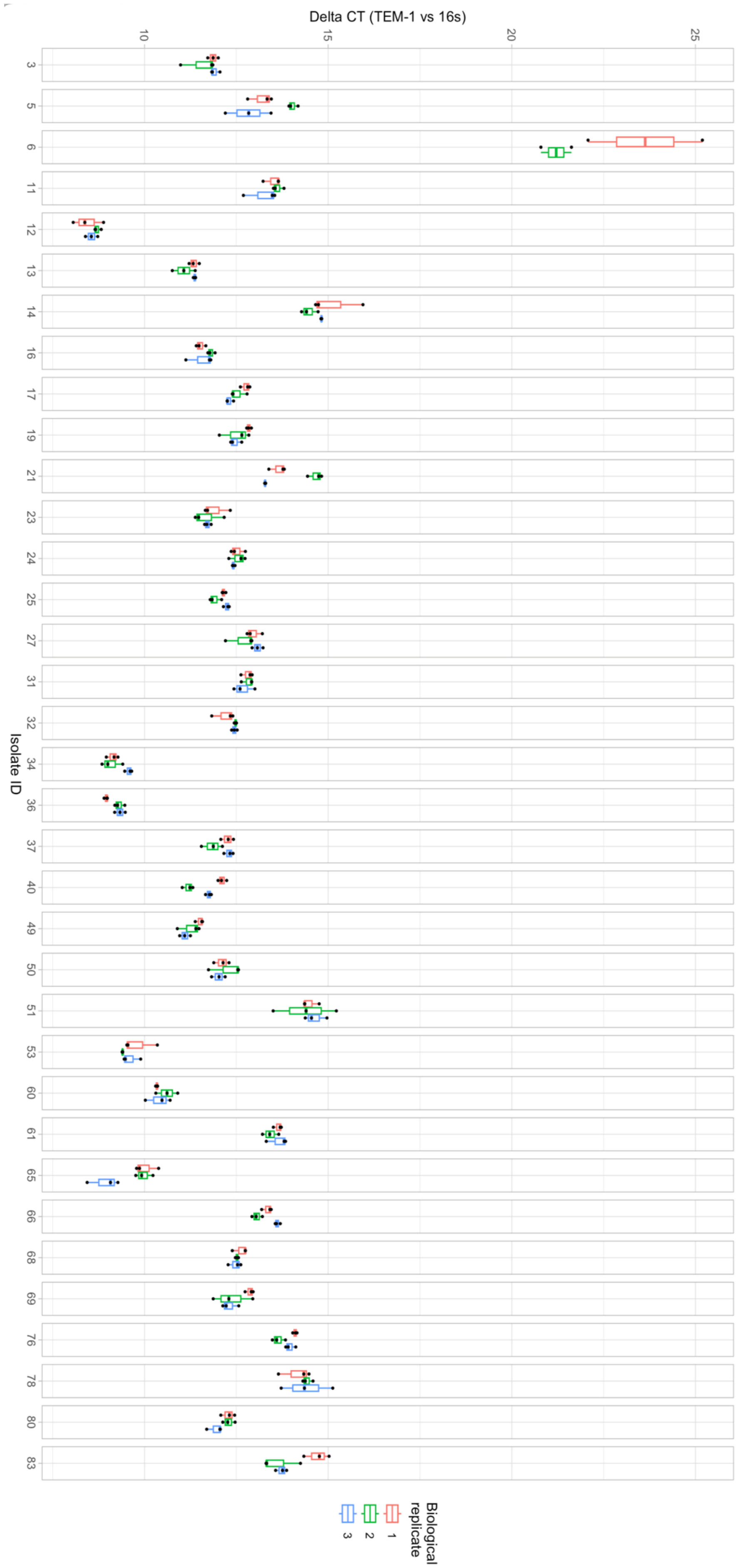

Figure S7. qPCR biological replicates. qPCR replicates by isolate (batch 1).

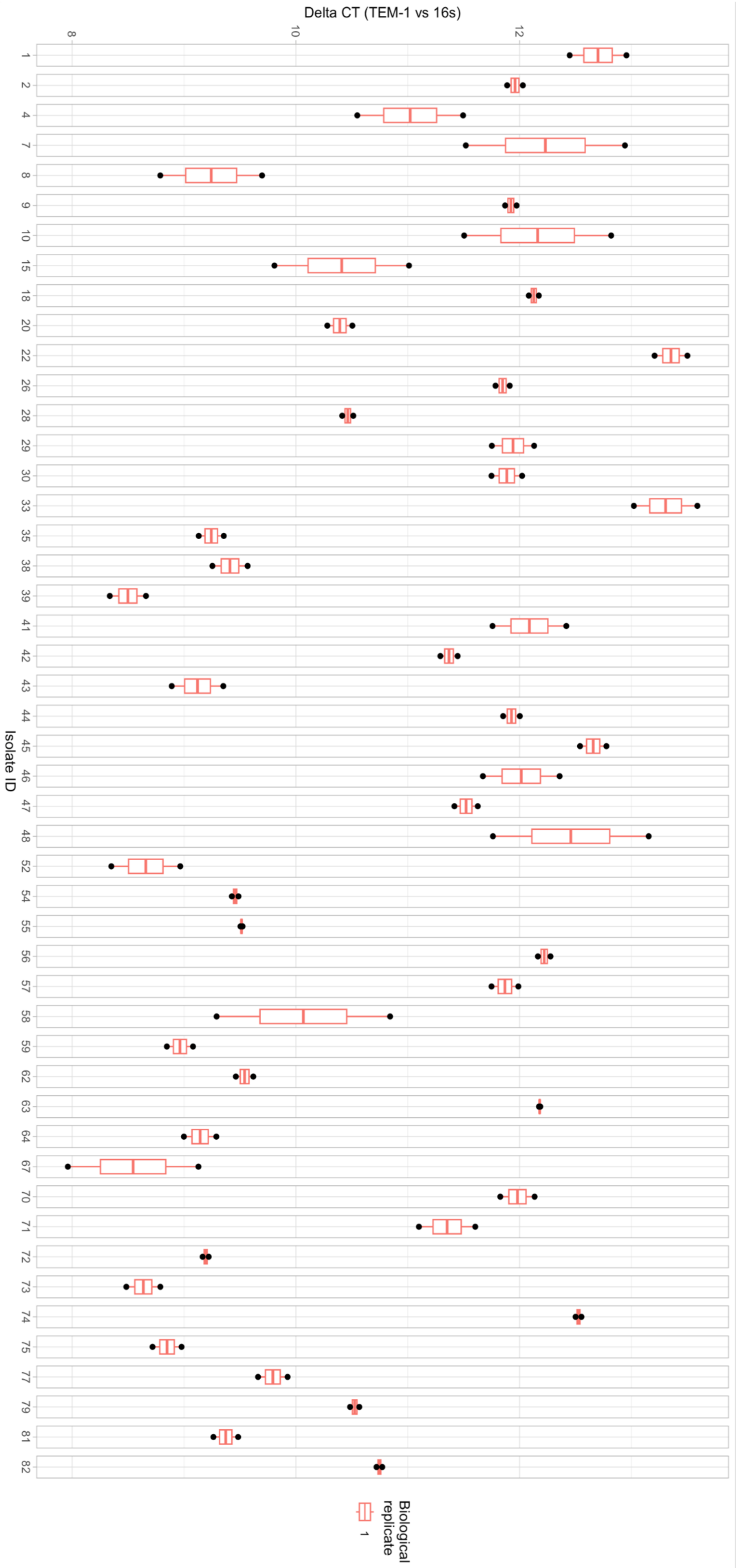

Figure S8. qPCR biological replicates. qPCR replicates by isolate (batch 2).

Supplementary Methods - genomic data

Genome assembly

We first performed quality control on both the short- and long-read sets (see readQC.sh). For the short-reads, we ran fastp (v. 0.23.4) with default parameters to remove any unpaired short-reads. For the long-reads, we ran filtlong (v. 0.2.1) with default parameters except --min_length 1000 --keep_percent 95 .This removed any reads shorter than 1kbp and excluded the worst 5% of reads.

We then assembled the genomes using two methods:

1. **Flye.** We first built a long-read assembly graph with Flye (v. 2.9.2-b1786; default parameters except --plasmids --nano-raw; see runFlye.sh). Next, we polished the graph with long-reads using medaka (v. 1.8.0; default parameters except -m r941_min_high_g360). Medaka did not provide a model which perfectly matched our set-up (MinION with a R9.4.1 flow cell and Guppy [v. 3.84] in SUP mode), so we used r941_min_high_g360 as the closest match. Lastly, we polished the graph with short-reads. To do this, we first aligned our short-reads to the assembly with bwa (v. 0.7.17-r1188; index mode followed mem mode for both halves of the short-read sets; default parameters), then Polypolish (v. 0.5.0; polypolish_insert_filter.py followed by running with default parameters). Proceeding the polishing with a size filter excluded any excessive alignments. We polished with long-reads first because they are less likely to align to multiple positions. The short-read polishing then only made changes with unanimous agreement.
2. **Unicycler.** We produced a hybrid assembly with Unicycler (v. 0.5.0; see runUnicycler.sh). This method produces a short-read assembly graph with SPAdes (v. 3.15.5), and then uses the long-reads to bridge between contigs with miniasm (v. 0.3-r179) and Racon (v. 1.5.0). We ran Unicycler with default settings except --mode conservative min_component_size 500 --min_dead_end_size 500.

Next, we approximated the depth of the short- and long-reads by dividing the total size of each read set by 5Mbp, the average size of an *E. coli* genome (see approxDepth.py). Taking any value less than 50 to be 'shallow' and anything larger to be 'deep', 0.5% (3/548) had shallow short- and long-reads, 1.5% (8/548) had shallow short-reads and deep long-reads, 26.5% (145/548) has deep short-reads and shallow long-reads, and 71.5% (392/548) had deep short- and long-reads. We found that when short-reads were deep, regardless of long-read depth, the Unicycler method was better at recovering a "complete" assembly (all contigs circularised; 46.7% [251/537] for Unicycler versus 33.0% [177/537] for Flye), but the Flye method was better at circularising the chromosome (57.9% [311/537] for Unicycler versus 66.9% [359/537] for Flye).

For *n*=3 isolates (OXEC-60, OXEC-247, and OXEC-273), we had to subsample their long-reads due to memory constraints. Before, isolates OXEC-60, OXEC-247, and OXEC-273 had approximate long-read depths of 519, 485, and 561, respectively. We used Rasusa (v. 0.7.1) with default parameters except --coverage 200 --genome-size 5mb to reduce them to an approximate depth of 200.

Long-read-first assemblies are better at resolving repetitive regions in genomes than short-read-first assemblies. Also, for this study, having a complete assembly was preferable to only having a circularised chromosome. This determined our order of preference in the final assembly choice: 1st choice a complete Flye assembly (177/548); 2nd choice a complete Unicycler assembly (148/548); 3rd choice a circularised-chromosome Flye assembly (85/548); 4th choice a circularised-chromosome Unicycler assembly (22/548). In total, we kept 78.8% (432/548) of genome assemblies, discarding the remaining 21.2% (116/548).

Dataset curation

Firstly, we observed one isolate (OXEC-153) for which the assembly chromosome length was 3,560,788bp < 4.5Mbp, which we removed, leaving *n*=431 assemblies. Then, to identify $bla_{\text{TEM-1}}$ and other AMR genes, all assemblies were annotated with NCBIAMRFinder (v. 3.11.26 and database v. 2023-11-15.1) with default parameters except --plus --organism Escherichia. Alongside, we validated the presence of $bla_{\text{TEM-1}}$ using tblastn (v. 2.15.0+) with the NCBI Reference Gene Catalog TEM-1 RefSeq protein WP_000027057.1 and 100% amino acid identity. At this stage, *n*=42 assemblies were found to not carry $bla_{\text{TEM-1}}$, and a further *n*=12 were found to carry additional beta-lactamases, leaving a total of *n*=377 assemblies in our final dataset.

Validating plasmid contigs

To confirm the origin of the non-chromosomal contigs, we used Mash screen (v. 2.3) to score their containment in plasmids from PLSDB (v. 2023_06_23_v2), a curated database of 50,554 plasmid sequences curated from NCBI (see screenPlsdb.sh). For each of our assembly contigs, we kept the top hit.

Supplementary Methods - modelling

Single phenotype model specifications

Both of the genotype-phenotype models are generalised linear models with mixed effects (GLMMs), structured as

$$\boldsymbol{Y} = X\boldsymbol{\beta} + \sum_i Z_i \boldsymbol{u_i} + \boldsymbol{\varepsilon}$$

where $\boldsymbol{Y}$ is the response vector, is the X design matrix for the fixed effects, $\boldsymbol{\beta}$ is the fixed effects coefficients, Z_i is the design matrix for the random effect i, is $\boldsymbol{u_i}$ the random effects coefficients, and $\boldsymbol{\varepsilon}$ is the residual errors.

In the *bla*TEM-1 expression model, the response variable $\boldsymbol{Y}$ is assumed to follow a multivariate normal distribution

$$\boldsymbol{Y} \sim \mathrm{N}(X\boldsymbol{\beta} + \Sigma_i Z_i \boldsymbol{u_i}, \sigma^2 I)$$

where σ^2 is the residual variance. The fixed effects are assumed to follow a multivariate normal distribution

$$\boldsymbol{\beta} \sim \mathrm{N}(\boldsymbol{\mu_\beta}, \boldsymbol{\sigma^2_\beta})$$

The random effects for qPCR replicates are given by $Z_1\boldsymbol{u_1}$ and

$$\boldsymbol{u_1} \sim \mathrm{N}\left(\boldsymbol{0}, \sigma^2_{u_1} I\right)$$

The phylogenetic random effects are given by $Z_2\boldsymbol{u_2}$ and

$$\boldsymbol{u_2} \sim \mathrm{N}\left(\boldsymbol{0}, \sigma^2_{u_2} A^{-1}\right)$$

where A^{-1} is the inverse of the phylogenetic relationship matrix (see later). The residuals $\boldsymbol{\varepsilon}$ are assumed to follow a multivariate normal distribution

$$\boldsymbol{\varepsilon} \sim \mathrm{N}(\boldsymbol{0}, \sigma^2 I)$$

The co-amoxiclav MIC model is specified similarly, except there is no random effect for qPCR replicate, and $\boldsymbol{Y}$ is assumed to follow an ordinal distribution modelled through underlying continuous latent variables

$$Y_i^* \sim \mathrm{N}(\mu_i, \sigma^2)$$

and the observed ordinal response Y_i is determined by cutpoints θ_i applied to Y_i^*

$$Y_i = \begin{cases} 1 & \text{if } Y_i^* \leq \theta_1 \\ 2 & \text{if } \theta_1 < Y_i^* \leq \theta_2 \\ \vdots & \\ k & \text{if } \theta_{k-1} < Y_i^* \end{cases}$$

Parameter estimation

We used Markov Chain Monte Carlo (MCMC) to sample posterior distributions for the fixed effects, random effects, variance components, and for the ordinal model, cutpoints. Fixed effects use normal priors $\mathrm{N}(\mathbf{0}, 10^{10} \times \boldsymbol{I})$. Priors for the variance components of qPCR replicate random effect and residual errors were Inverse-Wishart$(1, 0.02)$. For the phylogenetic random effect variance, to improve the MCMC mixing, we defined it as the product $u_i = \alpha\eta_i$ where $\eta_i \sim \mathrm{N}(0, V_\eta)$, which yields the two priors $\alpha \sim \mathrm{N}(0, V_\alpha)$ and $V_\eta \sim$ Inverse-Gamma(V, ν). We set $\alpha \sim \mathrm{N}(0, 1000)$ and Inverse-Gamma$(0.001,\ 0.001)$. In the co-amoxiclav MIC model, we fixed the residual variance at 1.

For both models, we ran two chains for 10 million iterations with 10% burn-in and a thinning interval of 100. To test for convergence, we calculated the Gelman-Rubin statistic for each model to confirm it was invariably 1 for all parameters. It assumes that if the chains have converged, the between-chain variance should be similar to the within-chain variance. We also verified that effective sample sizes were comparable between chains. We calculated the autocorrelation function for all chains and visually inspected the trace plots for global trends. For all parameters, we calculated the posterior means and 95% high density intervals.

Parameter values reported in the manuscript refer to the first chain.

For a parameter β, $p_{\mathrm{MCMC}}(\beta) = 2 \cdot \min\big(\mathbb{P}(\beta > 0), \mathbb{P}(\beta < 0)\big)$ and measures the probability that β is in the more extreme tail of its posterior distribution. If β were truly centred around zero, we would expect $\mathbb{P}(\beta > 0) \approx \mathbb{P}(\beta < 0) \approx 0.5$.

Phylogeny as a random effect

Here we assume that more closely related isolates have similar responses due to their shared evolutionary history. With the *E. coli* chromosomal phylogeny, we took the midpoint root, then coerced the tree into ultrametricity using a penalised likelihood method which assumed that assumed that rates of branch evolution were correlated. Then, taking the transformed phylogeny as a variance-covariance matrix A, we inverted it to generate the precision matrix A^{-1}. Using the precision matrix is generally more numerically stable.

Best Linear Unbiased Predictors (BLUPs) represent the deviation of individual isolates from the average effect across the entire phylogeny. The total variance of all BLUPs is the phylogenetic random effect variance.

***bla*$_{\mathbf{TEM\text{-}1}}$ expression model outputs**

```
> summary(chain.1)

 Iterations = 1000001:9999901
```

```
 Thinning interval  = 100
 Sample size  = 90000 

 DIC: -84.31782 

 G-structure:  ~phylo

      post.mean  l-95% CI u-95% CI eff.samp
phylo   0.06803 6.734e-12   0.2066    48985

               ~isolate.assembly

                 post.mean l-95% CI u-95% CI eff.samp
isolate.assembly    0.1498  0.07779   0.2272    76382

 R-structure:  ~units

      post.mean l-95% CI u-95% CI eff.samp
units   0.03356  0.02711  0.04045    90000

 Location effects: exp.scaled ~ pos1.bool * pos2.bool + 
contig.copy.number.scaled 

                            post.mean  l-95% CI  u-95% CI 
eff.samp  pMCMC    
(Intercept)                  0.297258  0.031125  0.585104    
87300 0.0245 *  
pos1.boolTRUE               -1.713268 -2.079701 -1.343259    
90000 <1e-05 ***
pos2.boolTRUE               -0.313958 -0.570731 -0.042875    
90000 0.0202 *  
contig.copy.number.scaled   -0.116605 -0.242749  0.007042    
87138 0.0679 .  
pos1.boolTRUE:pos2.boolTRUE  0.325914 -0.143755  0.811333    
88934 0.1779    
---
Signif. codes:  0 '***' 0.001 '**' 0.01 '*' 0.05 '.' 0.1 ' ' 1

> autocorr.diag(chain.1$VCV)
               phylo isolate.assembly         units
Lag 0    1.000000000     1.0000000000  1.0000000000
Lag 100  0.147970235     0.0373112895  0.0019415565
Lag 500  0.027018030     0.0048930316 -0.0007288071
Lag 1000 0.005572987    -0.0014661450  0.0003393882
Lag 5000 0.000294595     0.0007567522  0.0024936229

> plot(chain.1)
```

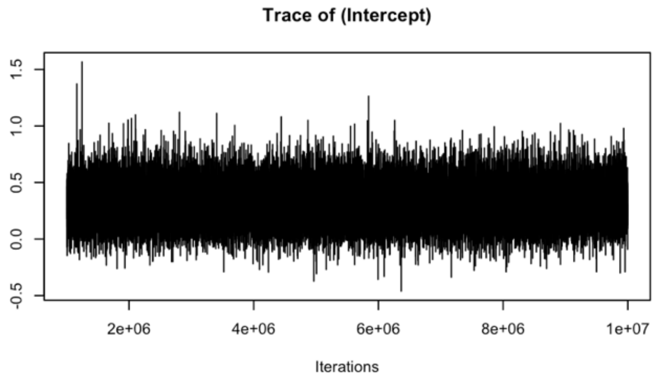
Trace of (Intercept)
Iterations
2e+06 4e+06 6e+06 8e+06 1e+07
-0.5 0.0 0.5 1.0 1.5

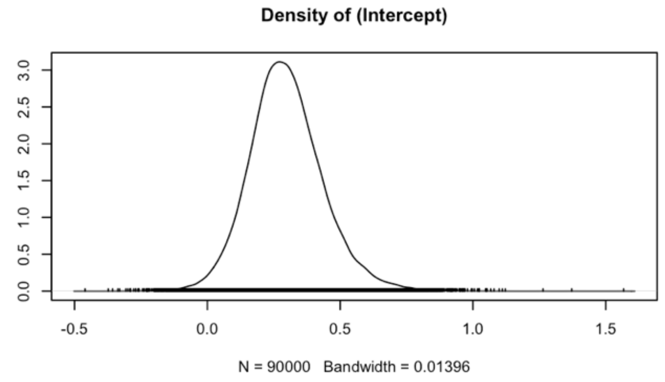
Density of (Intercept)
N = 90000 Bandwidth = 0.01396
-0.5 0.0 0.5 1.0 1.5
0.0 0.5 1.0 1.5 2.0 2.5 3.0

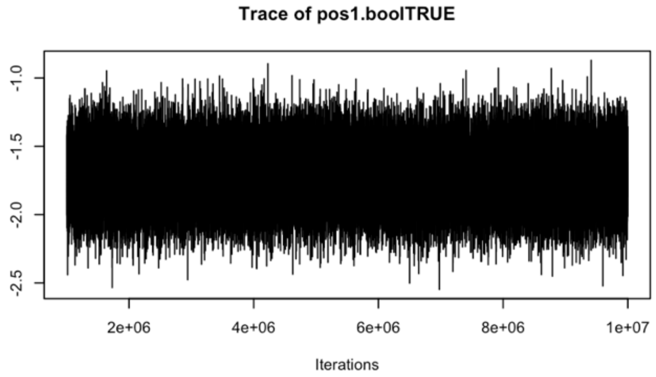
Trace of pos1.boolTRUE
Iterations
2e+06 4e+06 6e+06 8e+06 1e+07
-2.5 -2.0 -1.5 -1.0

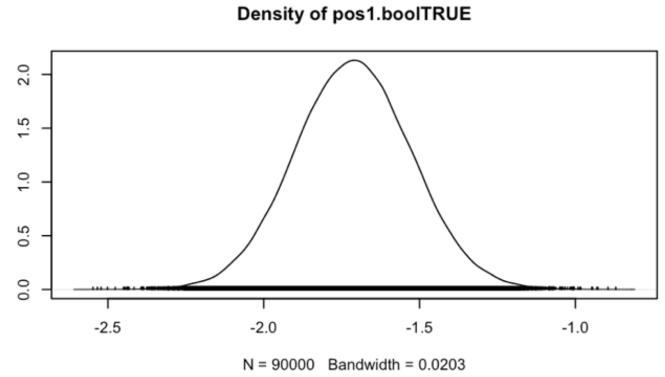
Density of pos1.boolTRUE
N = 90000 Bandwidth = 0.0203
-2.5 -2.0 -1.5 -1.0
0.0 0.5 1.0 1.5 2.0

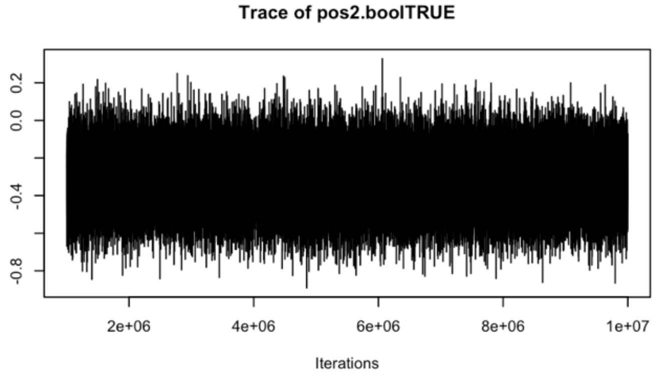
Trace of pos2.boolTRUE
Iterations
2e+06 4e+06 6e+06 8e+06 1e+07
-0.8 -0.4 0.0 0.2

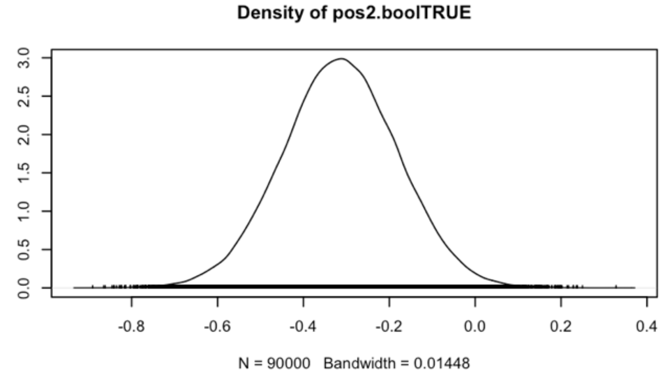
Density of pos2.boolTRUE
N = 90000 Bandwidth = 0.01448
-0.8 -0.6 -0.4 -0.2 0.0 0.2 0.4
0.0 0.5 1.0 1.5 2.0 2.5 3.0

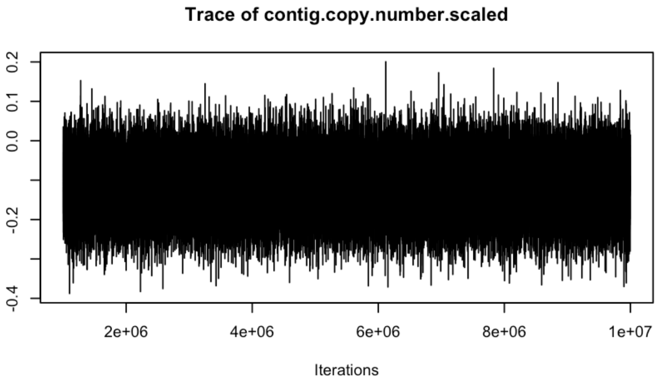
Trace of contig.copy.number.scaled
Iterations
2e+06 4e+06 6e+06 8e+06 1e+07
-0.4 -0.2 0.0 0.1 0.2

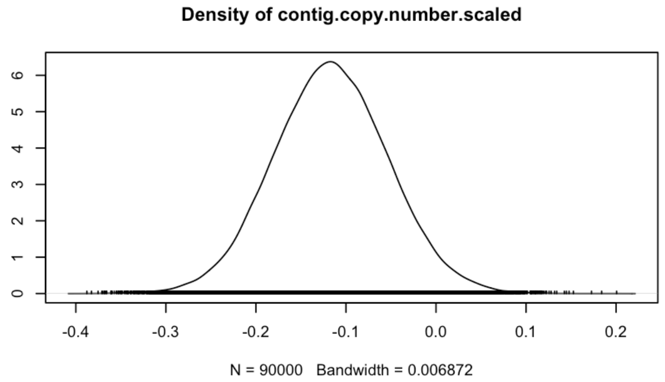
Density of contig.copy.number.scaled
N = 90000 Bandwidth = 0.006872
-0.4 -0.3 -0.2 -0.1 0.0 0.1 0.2
0 1 2 3 4 5 6

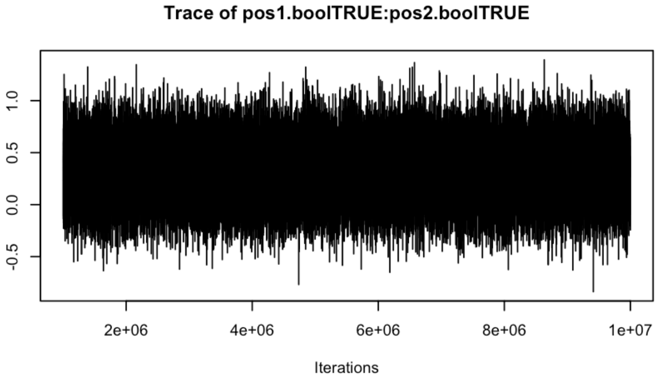
Trace of pos1.boolTRUE:pos2.boolTRUE
Iterations
2e+06 4e+06 6e+06 8e+06 1e+07
-0.5 0.0 0.5 1.0

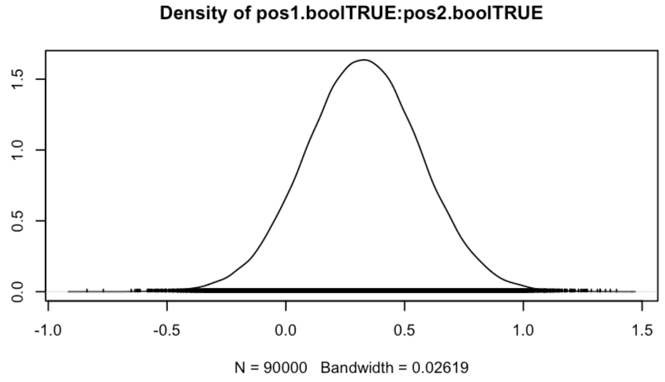
Density of pos1.boolTRUE:pos2.boolTRUE
N = 90000 Bandwidth = 0.02619
-1.0 -0.5 0.0 0.5 1.0 1.5
0.0 0.5 1.0 1.5

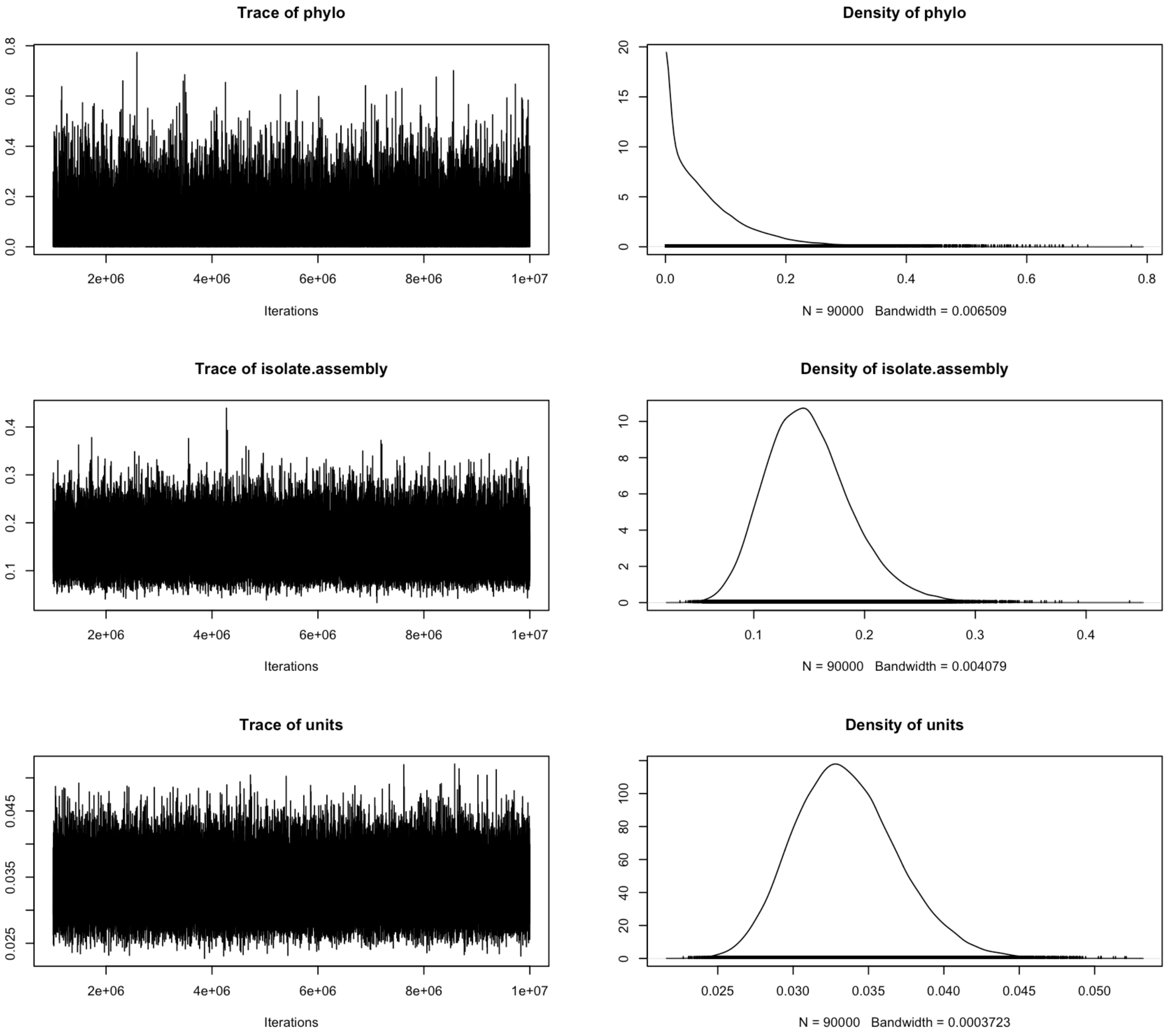


```
> summary(chain.2)

 Iterations = 1000001:9999901
 Thinning interval  = 100
 Sample size  = 90000

 DIC: -84.35054

 G-structure:  ~phylo

      post.mean  l-95% CI u-95% CI eff.samp
phylo   0.06815 3.565e-10   0.2062    48048

               ~isolate.assembly

                 post.mean l-95% CI u-95% CI eff.samp
isolate.assembly    0.1499  0.07934   0.2281    74765

 R-structure:  ~units
```

```
      post.mean l-95% CI u-95% CI eff.samp
units   0.03355   0.0272  0.04045    91734

 Location effects: exp.scaled ~ pos1.bool * pos2.bool +
contig.copy.number.scaled

                            post.mean  l-95% CI  u-95% CI
eff.samp  pMCMC
(Intercept)                  0.296815  0.038728  0.590741
88946 0.0247 *
pos1.boolTRUE               -1.713476 -2.083985 -1.344730
90000 <1e-05 ***
pos2.boolTRUE               -0.313962 -0.585371 -0.054027
90000 0.0210 *
contig.copy.number.scaled   -0.117043 -0.242901  0.008085
87127 0.0684 .
pos1.boolTRUE:pos2.boolTRUE  0.326406 -0.146791  0.812865
90000 0.1787
---
Signif. codes:  0 '***' 0.001 '**' 0.01 '*' 0.05 '.' 0.1 ' ' 1

> autocorr.diag(chain.2$VCV)
               phylo isolate.assembly         units
Lag 0    1.000000000     1.0000000000  1.0000000000
Lag 100  0.144760011     0.0425125177 -0.0040162838
Lag 500  0.027608837     0.0107320749  0.0041080909
Lag 1000 0.007409151    -0.0038731455 -0.0026025067
Lag 5000 0.002530502     0.0006590854  0.0004411566

> plot(chain.2)
```

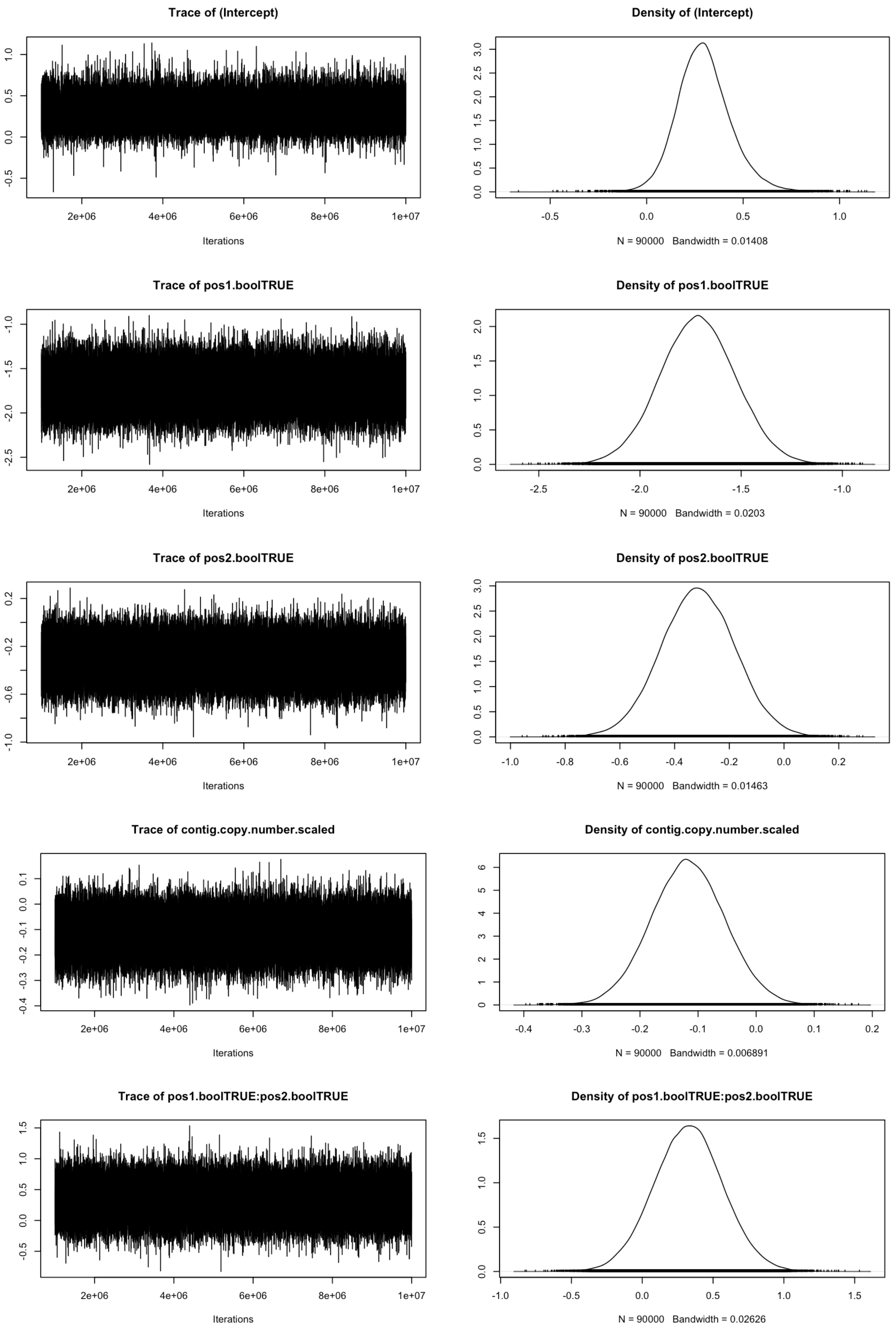
Trace of (Intercept)
Iterations
Density of (Intercept)
N = 90000 Bandwidth = 0.01408
Trace of pos1.boolTRUE
Iterations
Density of pos1.boolTRUE
N = 90000 Bandwidth = 0.0203
Trace of pos2.boolTRUE
Iterations
Density of pos2.boolTRUE
N = 90000 Bandwidth = 0.01463
Trace of contig.copy.number.scaled
Iterations
Density of contig.copy.number.scaled
N = 90000 Bandwidth = 0.006891
Trace of pos1.boolTRUE:pos2.boolTRUE
Iterations
Density of pos1.boolTRUE:pos2.boolTRUE
N = 90000 Bandwidth = 0.02626

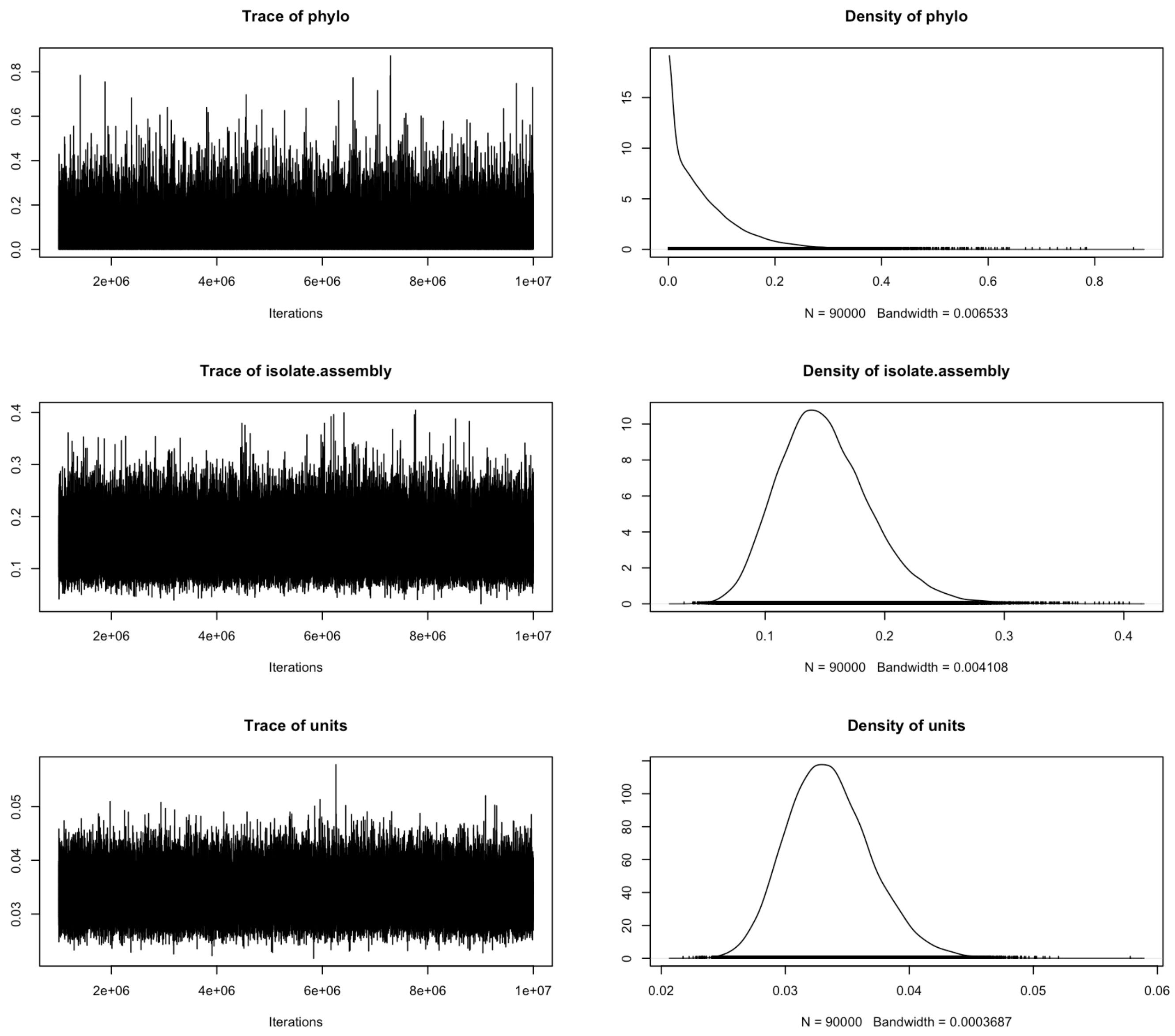


```
> mclist <- mcmc.list(chain.1$Sol, chain.2$Sol)
> gelman.diag(mclist)
Potential scale reduction factors:

                              Point est. Upper C.I.
(Intercept)                            1          1
pos1.boolTRUE                          1          1
pos2.boolTRUE                          1          1
contig.copy.number.scaled              1          1
pos1.boolTRUE:pos2.boolTRUE            1          1
phylo.542                              1          1
phylo.414                              1          1
phylo.236                              1          1
phylo.415                              1          1
phylo.476                              1          1
phylo.257                              1          1
phylo.103                              1          1
phylo.285                              1          1
phylo.284                              1          1
phylo.410                              1          1
```

phylo.344	1	1
phylo.429	1	1
phylo.322	1	1
phylo.262	1	1
phylo.387	1	1
phylo.158	1	1
phylo.365	1	1
phylo.267	1	1
phylo.477	1	1
phylo.197	1	1
phylo.41	1	1
phylo.408	1	1
phylo.292	1	1
phylo.1	1	1
phylo.480	1	1
phylo.423	1	1
phylo.247	1	1
phylo.353	1	1
phylo.532	1	1
phylo.203	1	1
phylo.534	1	1
phylo.192	1	1
phylo.334	1	1
phylo.486	1	1
phylo.541	1	1
phylo.462	1	1
phylo.93	1	1
phylo.230	1	1
phylo.457	1	1
phylo.176	1	1
phylo.75	1	1
phylo.296	1	1
phylo.442	1	1
phylo.495	1	1
phylo.116	1	1
phylo.233	1	1
phylo.83	1	1
phylo.508	1	1
phylo.386	1	1
phylo.59	1	1
phylo.490	1	1
phylo.125	1	1
phylo.69	1	1
phylo.340	1	1
phylo.25	1	1
phylo.546	1	1
phylo.279	1	1
phylo.485	1	1
phylo.38	1	1
phylo.238	1	1
phylo.544	1	1

phylo.314	1	1
phylo.345	1	1
phylo.73	1	1
phylo.190	1	1
phylo.107	1	1
phylo.440	1	1
isolate.assembly.1	1	1
isolate.assembly.25	1	1
isolate.assembly.38	1	1
isolate.assembly.41	1	1
isolate.assembly.59	1	1
isolate.assembly.69	1	1
isolate.assembly.73	1	1
isolate.assembly.75	1	1
isolate.assembly.83	1	1
isolate.assembly.93	1	1
isolate.assembly.103	1	1
isolate.assembly.107	1	1
isolate.assembly.116	1	1
isolate.assembly.125	1	1
isolate.assembly.158	1	1
isolate.assembly.176	1	1
isolate.assembly.190	1	1
isolate.assembly.192	1	1
isolate.assembly.197	1	1
isolate.assembly.203	1	1
isolate.assembly.230	1	1
isolate.assembly.233	1	1
isolate.assembly.236	1	1
isolate.assembly.238	1	1
isolate.assembly.247	1	1
isolate.assembly.257	1	1
isolate.assembly.262	1	1
isolate.assembly.267	1	1
isolate.assembly.279	1	1
isolate.assembly.284	1	1
isolate.assembly.285	1	1
isolate.assembly.292	1	1
isolate.assembly.296	1	1
isolate.assembly.314	1	1
isolate.assembly.322	1	1
isolate.assembly.334	1	1
isolate.assembly.340	1	1
isolate.assembly.344	1	1
isolate.assembly.345	1	1
isolate.assembly.353	1	1
isolate.assembly.365	1	1
isolate.assembly.386	1	1
isolate.assembly.387	1	1
isolate.assembly.408	1	1
isolate.assembly.410	1	1

```
isolate.assembly.414                 1          1
isolate.assembly.415                 1          1
isolate.assembly.423                 1          1
isolate.assembly.429                 1          1
isolate.assembly.440                 1          1
isolate.assembly.442                 1          1
isolate.assembly.457                 1          1
isolate.assembly.462                 1          1
isolate.assembly.476                 1          1
isolate.assembly.477                 1          1
isolate.assembly.480                 1          1
isolate.assembly.485                 1          1
isolate.assembly.486                 1          1
isolate.assembly.490                 1          1
isolate.assembly.495                 1          1
isolate.assembly.508                 1          1
isolate.assembly.532                 1          1
isolate.assembly.534                 1          1
isolate.assembly.541                 1          1
isolate.assembly.542                 1          1
isolate.assembly.544                 1          1
isolate.assembly.546                 1          1

Multivariate psrf

1
```

Co-amoxiclav MIC model outputs

```
> summary(chain.1)

 Iterations = 1000001:9999901
 Thinning interval  = 100
 Sample size  = 90000

 DIC:

 G-structure:  ~phylo

      post.mean l-95% CI u-95% CI eff.samp
phylo     2.798   0.7219    5.162    68932

 R-structure:  ~units

      post.mean l-95% CI u-95% CI eff.samp
units         1        1        1        0

 Location effects: coamox.mic ~
tem1.isolate.copy.number.scaled + tem1.isolate.scaled +
ampc.promoter.snv + promoter.snv
```

```
                                 post.mean l-95% CI u-95% CI
eff.samp  pMCMC
(Intercept)                         3.9156   2.9235   4.8511
64592 <1e-05 ***
tem1.isolate.copy.number.scaled     2.0694   1.3796   2.7576
85815 <1e-05 ***
tem1.isolate.scaledTRUE             0.9707   0.0239   1.9026
87875 0.0414 *
ampc.promoter.snvAGCTTCTAGGG        0.4353  -0.6341   1.4824
90000 0.4122
ampc.promoter.snvAGCTCCTAGGG        0.8712  -0.8929   2.5803
90000 0.3039
ampc.promoter.snvGATTCCTAGGG        0.8341  -1.3676   3.1105
91257 0.4402
promoter.snvCGGCGA                  0.1659  -0.3512   0.6906
90000 0.5313
promoter.snvTGGCGA                  6.0563   4.1520   8.0753
76514 <1e-05 ***
promoter.snvTGGCGG                  5.8662   3.8927   7.8659
81643 <1e-05 ***
---
Signif. codes:  0 '***' 0.001 '**' 0.01 '*' 0.05 '.' 0.1 ' ' 1

 Cutpoints:

                            post.mean l-95% CI u-95% CI
eff.samp
cutpoint.traitcoamox.mic.1     1.229   0.7684    1.703
42997
cutpoint.traitcoamox.mic.2     4.068   3.5414    4.560
26767
cutpoint.traitcoamox.mic.3     5.620   5.0844    6.120
27192
cutpoint.traitcoamox.mic.4     6.661   6.1714    7.000
28479

> autocorr.diag(chain.1$VCV)
                 phylo units
Lag 0     1.0000000000   NaN
Lag 100   0.0836490796   NaN
Lag 500   0.0040354588   NaN
Lag 1000  0.0005240083   NaN
Lag 5000 -0.0044962661   NaN

> plot(chain.1)
```

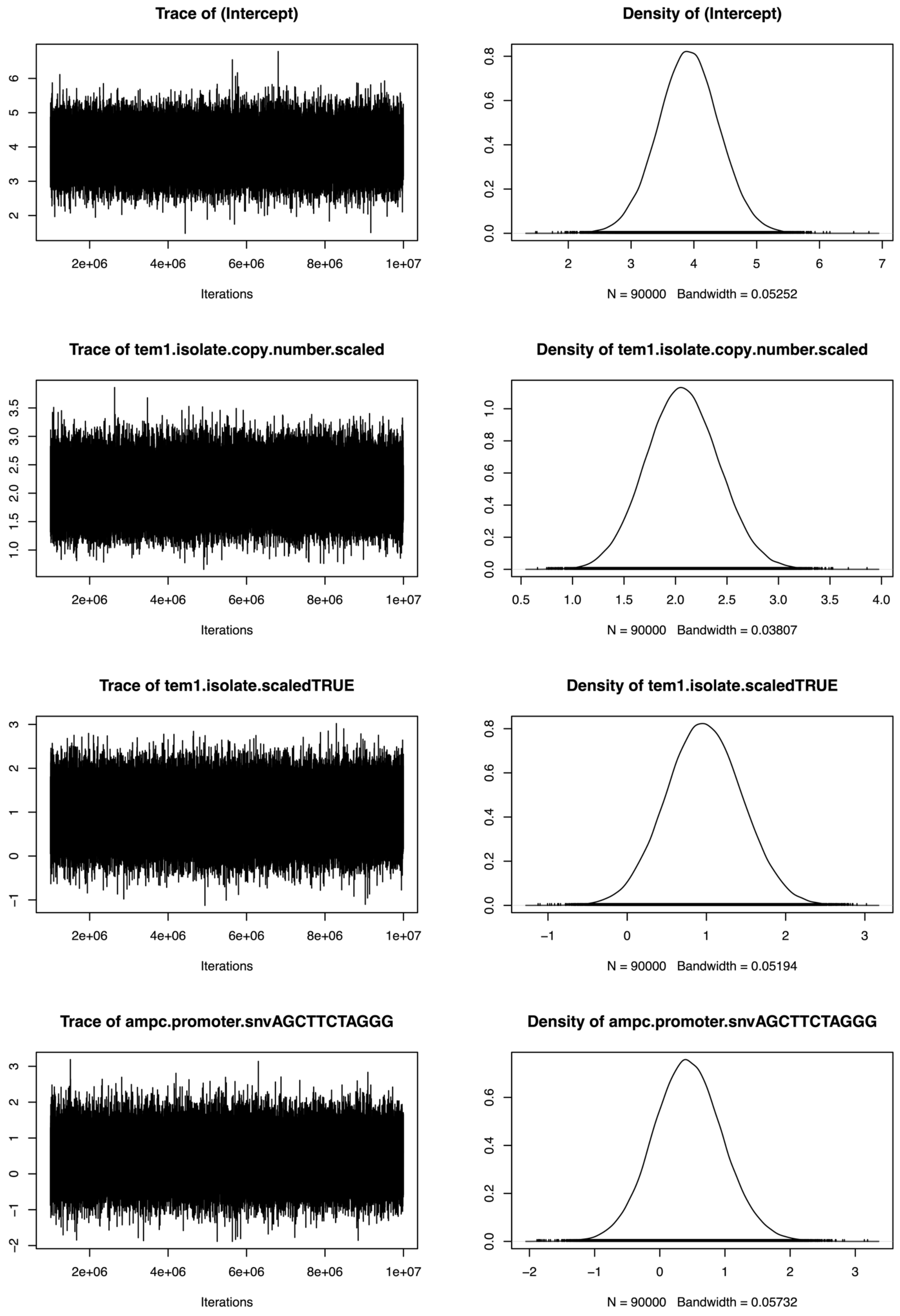
Trace of (Intercept)
Density of (Intercept)
Iterations
N = 90000 Bandwidth = 0.05252
Trace of tem1.isolate.copy.number.scaled
Density of tem1.isolate.copy.number.scaled
Iterations
N = 90000 Bandwidth = 0.03807
Trace of tem1.isolate.scaledTRUE
Density of tem1.isolate.scaledTRUE
Iterations
N = 90000 Bandwidth = 0.05194
Trace of ampc.promoter.snvAGCTTCTAGGG
Density of ampc.promoter.snvAGCTTCTAGGG
Iterations
N = 90000 Bandwidth = 0.05732

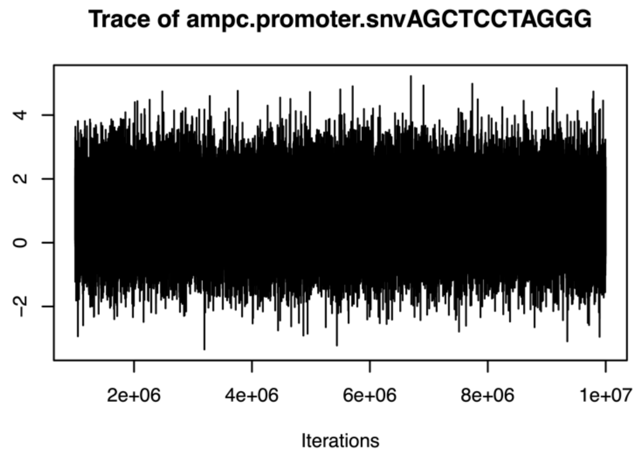
Trace of ampc.promoter.snvAGCTCCTAGGG
4
2
0
−2
2e+06
4e+06
6e+06
8e+06
1e+07
Iterations

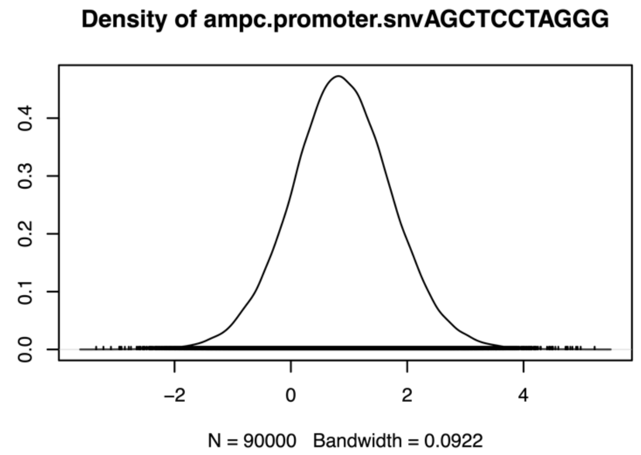
Density of ampc.promoter.snvAGCTCCTAGGG
0.4
0.3
0.2
0.1
0.0
−2
0
2
4
N = 90000 Bandwidth = 0.0922

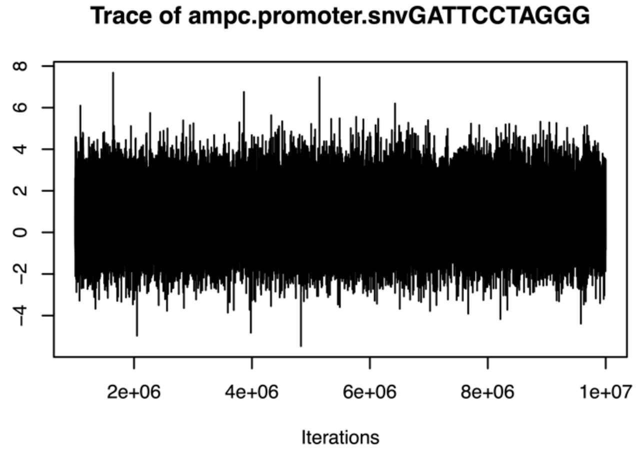
Trace of ampc.promoter.snvGATTCCTAGGG
8
6
4
2
0
−2
−4
2e+06
4e+06
6e+06
8e+06
1e+07
Iterations

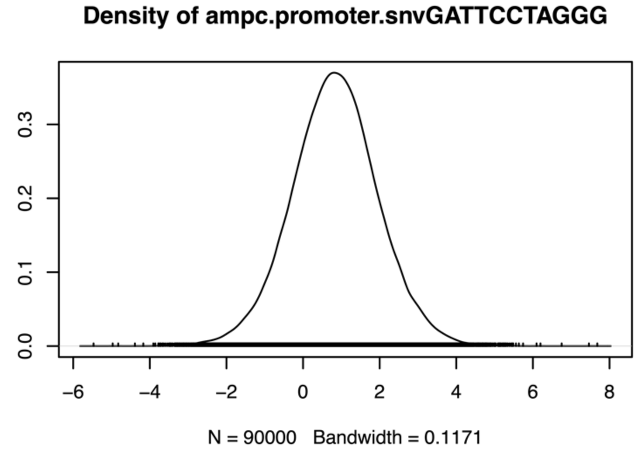
Density of ampc.promoter.snvGATTCCTAGGG
0.3
0.2
0.1
0.0
−6
−4
−2
0
2
4
6
8
N = 90000 Bandwidth = 0.1171

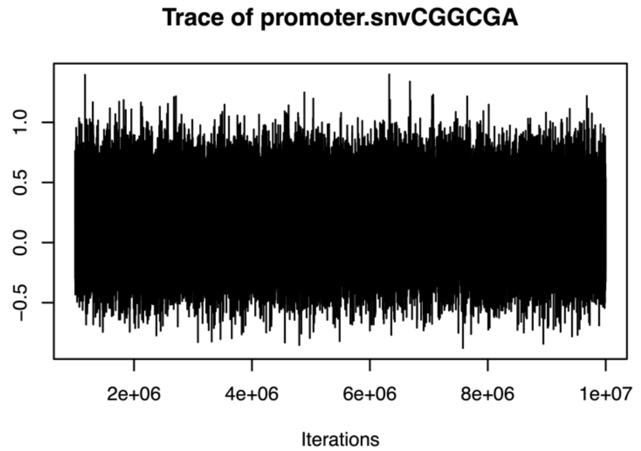
Trace of promoter.snvCGGCGA
1.0
0.5
0.0
−0.5
2e+06
4e+06
6e+06
8e+06
1e+07
Iterations

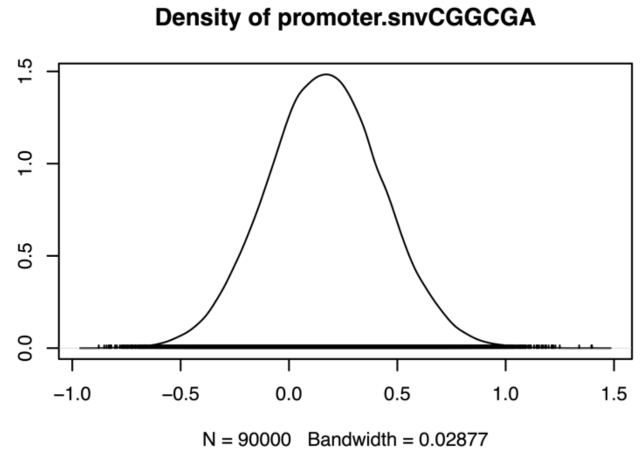
Density of promoter.snvCGGCGA
1.5
1.0
0.5
0.0
−1.0
−0.5
0.0
0.5
1.0
1.5
N = 90000 Bandwidth = 0.02877

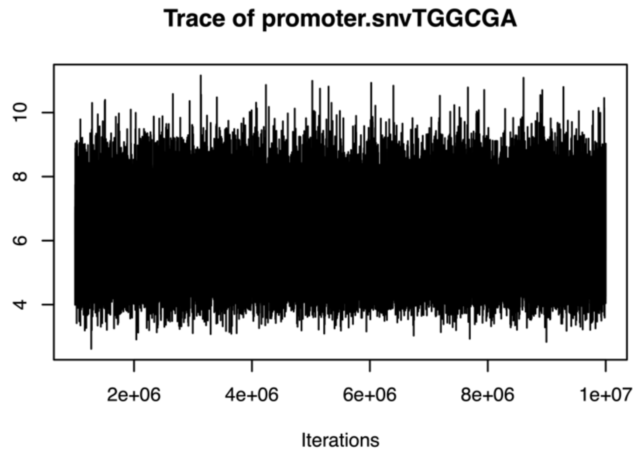
Trace of promoter.snvTGGCGA
10
8
6
4
2e+06
4e+06
6e+06
8e+06
1e+07
Iterations

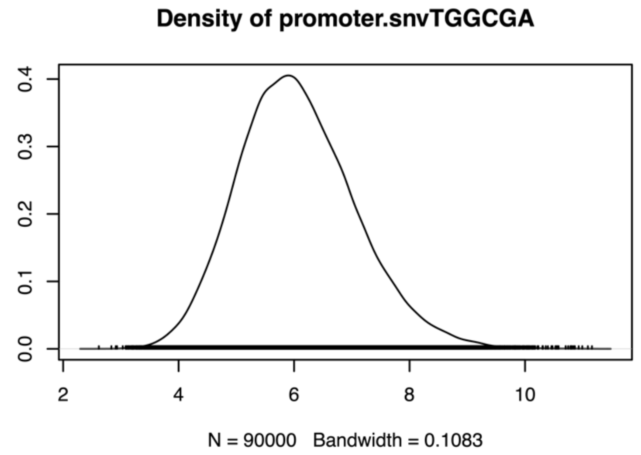
Density of promoter.snvTGGCGA
0.4
0.3
0.2
0.1
0.0
2
4
6
8
10
N = 90000 Bandwidth = 0.1083

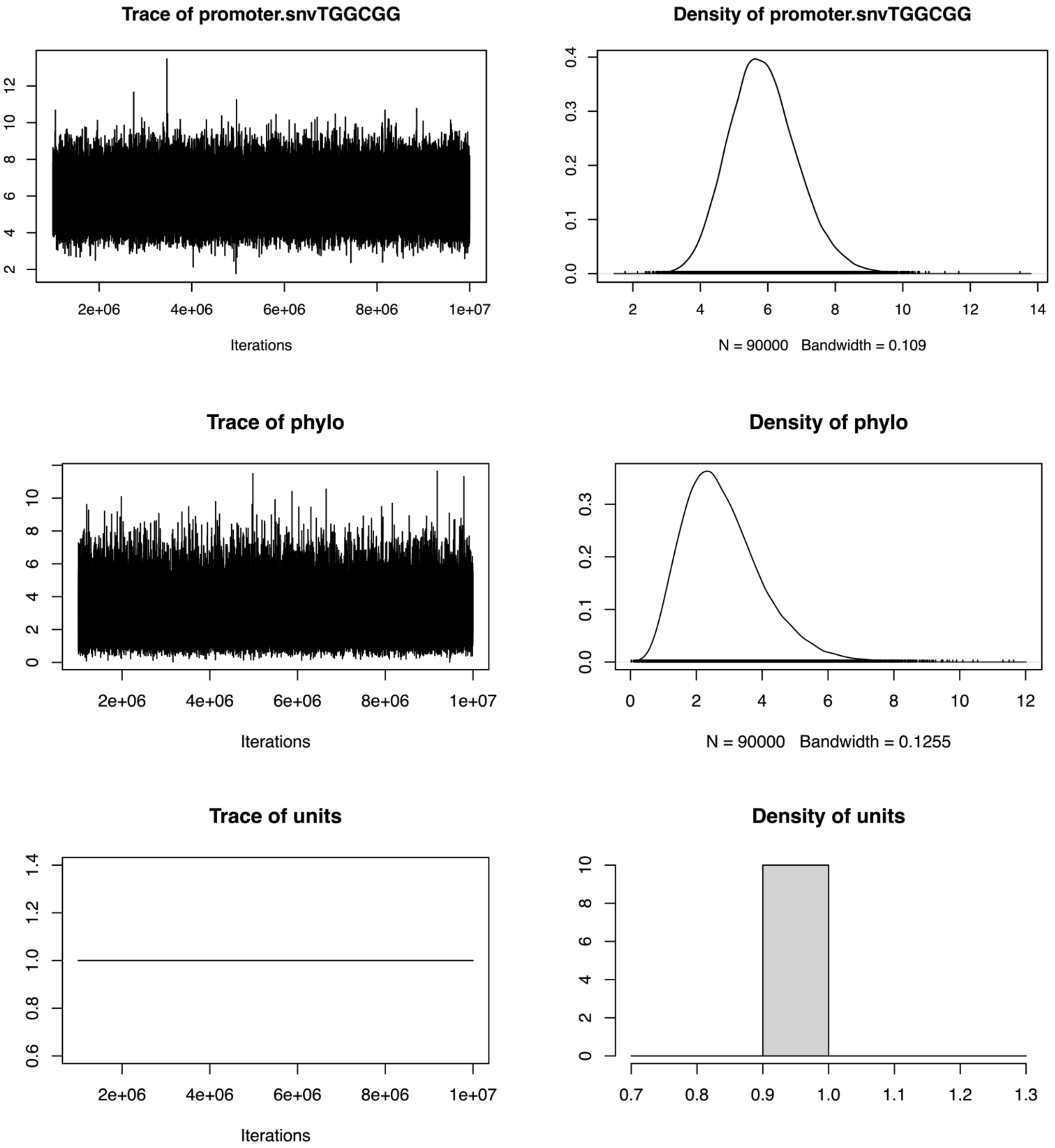
Trace of promoter.snvTGGCGG
Iterations
Density of promoter.snvTGGCGG
N = 90000   Bandwidth = 0.109
Trace of phylo
Iterations
Density of phylo
N = 90000   Bandwidth = 0.1255
Trace of units
Iterations
Density of units

```
> summary(chain.2)

 Iterations = 1000001:9999901
 Thinning interval  = 100
 Sample size  = 90000

 DIC:

 G-structure:  ~phylo

      post.mean l-95% CI u-95% CI eff.samp
phylo     2.791   0.7336    5.201    70573

 R-structure:  ~units

      post.mean l-95% CI u-95% CI eff.samp
units         1        1        1        0

 Location effects: coamox.mic ~
tem1.isolate.copy.number.scaled + tem1.isolate.scaled +
ampc.promoter.snv + promoter.snv

                                post.mean l-95% CI u-95% CI
eff.samp  pMCMC
(Intercept)                       3.91653  2.96791  4.88654
66646 <1e-05 ***
tem1.isolate.copy.number.scaled   2.06746  1.37473  2.75216
88094 <1e-05 ***
tem1.isolate.scaledTRUE           0.97165  0.02865  1.90532
91323 0.0418 *
ampc.promoter.snvAGCTTCTAGGG      0.43186 -0.59461  1.51426
88949 0.4130
ampc.promoter.snvAGCTCCTAGGG      0.86712 -0.89686  2.58517
90000 0.3089
ampc.promoter.snvGATTCCTAGGG      0.83898 -1.39385  3.08294
88185 0.4360
promoter.snvCGGCGA                0.16523 -0.36323  0.67718
90000 0.5318
promoter.snvTGGCGA                6.05424  4.15254  8.04872
77248 <1e-05 ***
promoter.snvTGGCGG                5.87050  3.94726  7.94549
80392 <1e-05 ***
---
Signif. codes:  0 '***' 0.001 '**' 0.01 '*' 0.05 '.' 0.1 ' ' 1

 Cutpoints:

                           post.mean l-95% CI u-95% CI
eff.samp
```

```
cutpoint.traitcoamox.mic.1     1.228   0.7657    1.692
46989
cutpoint.traitcoamox.mic.2     4.066   3.5388    4.556
30825
cutpoint.traitcoamox.mic.3     5.617   5.0824    6.114
30368
cutpoint.traitcoamox.mic.4     6.656   6.1694    7.000
31735

> autocorr.diag(chain.2$VCV)
               phylo units
Lag 0    1.000000000   NaN
Lag 100  0.082454914   NaN
Lag 500  0.002508777   NaN
Lag 1000 0.003659047   NaN
Lag 5000 0.004916941   NaN

> plot(chain.2)
```

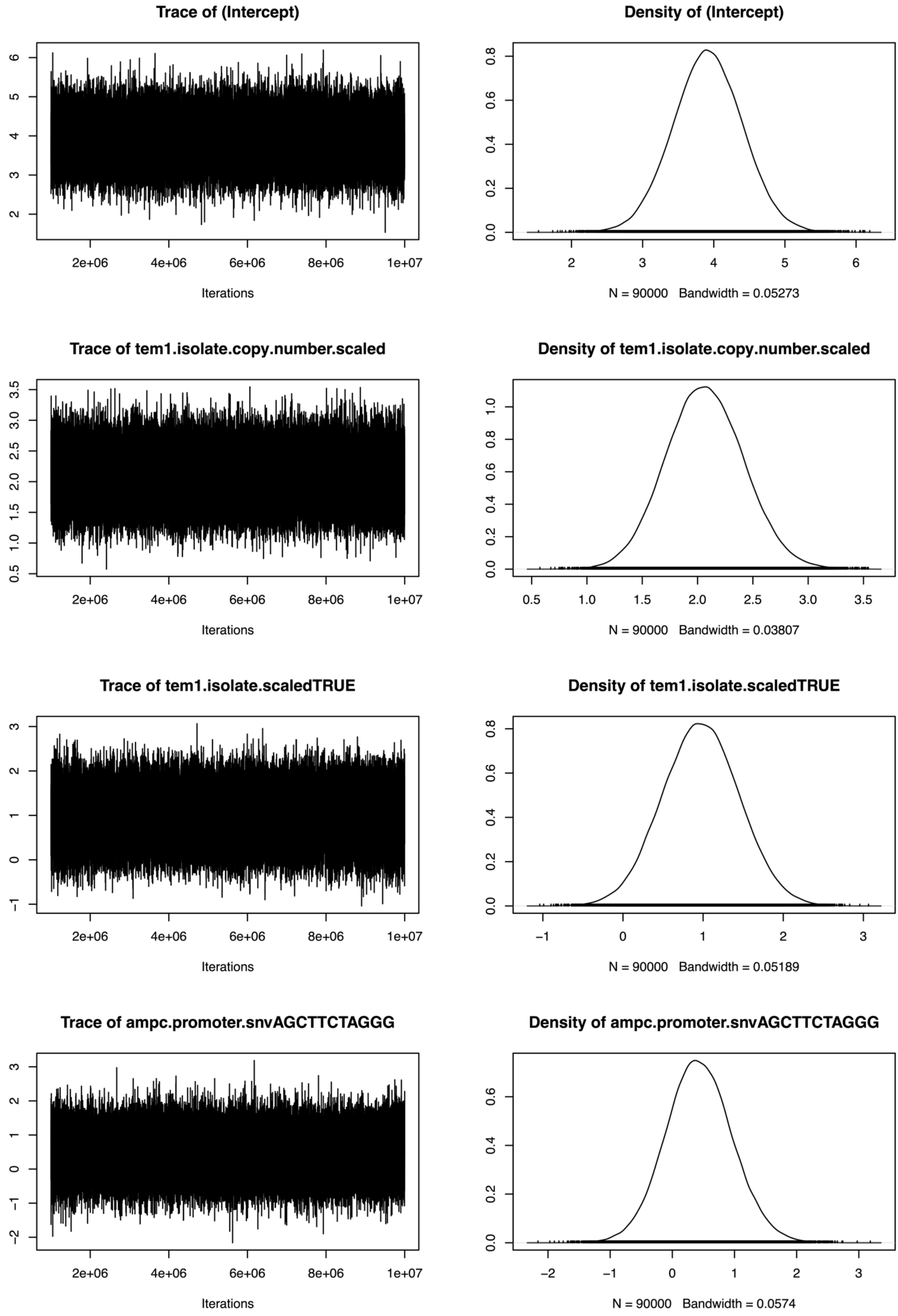
Trace of (Intercept)
Iterations
Density of (Intercept)
N = 90000   Bandwidth = 0.05273
Trace of tem1.isolate.copy.number.scaled
Iterations
Density of tem1.isolate.copy.number.scaled
N = 90000   Bandwidth = 0.03807
Trace of tem1.isolate.scaledTRUE
Iterations
Density of tem1.isolate.scaledTRUE
N = 90000   Bandwidth = 0.05189
Trace of ampc.promoter.snvAGCTTCTAGGG
Iterations
Density of ampc.promoter.snvAGCTTCTAGGG
N = 90000   Bandwidth = 0.0574

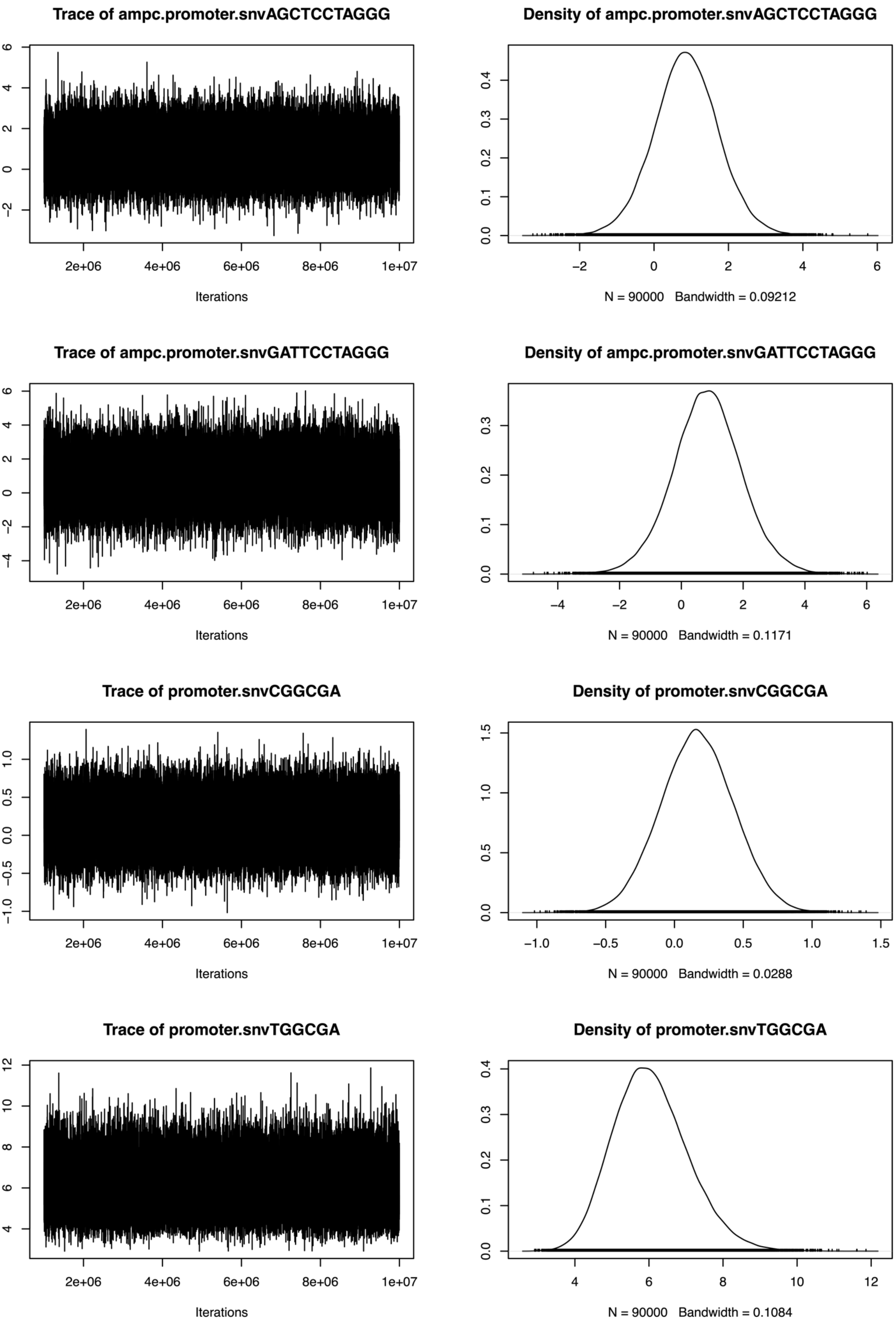

Trace of ampc.promoter.snvAGCTCCTAGGG
Iterations
Density of ampc.promoter.snvAGCTCCTAGGG
N = 90000 Bandwidth = 0.09212
Trace of ampc.promoter.snvGATTCCTAGGG
Iterations
Density of ampc.promoter.snvGATTCCTAGGG
N = 90000 Bandwidth = 0.1171
Trace of promoter.snvCGGCGA
Iterations
Density of promoter.snvCGGCGA
N = 90000 Bandwidth = 0.0288
Trace of promoter.snvTGGCGA
Iterations
Density of promoter.snvTGGCGA
N = 90000 Bandwidth = 0.1084

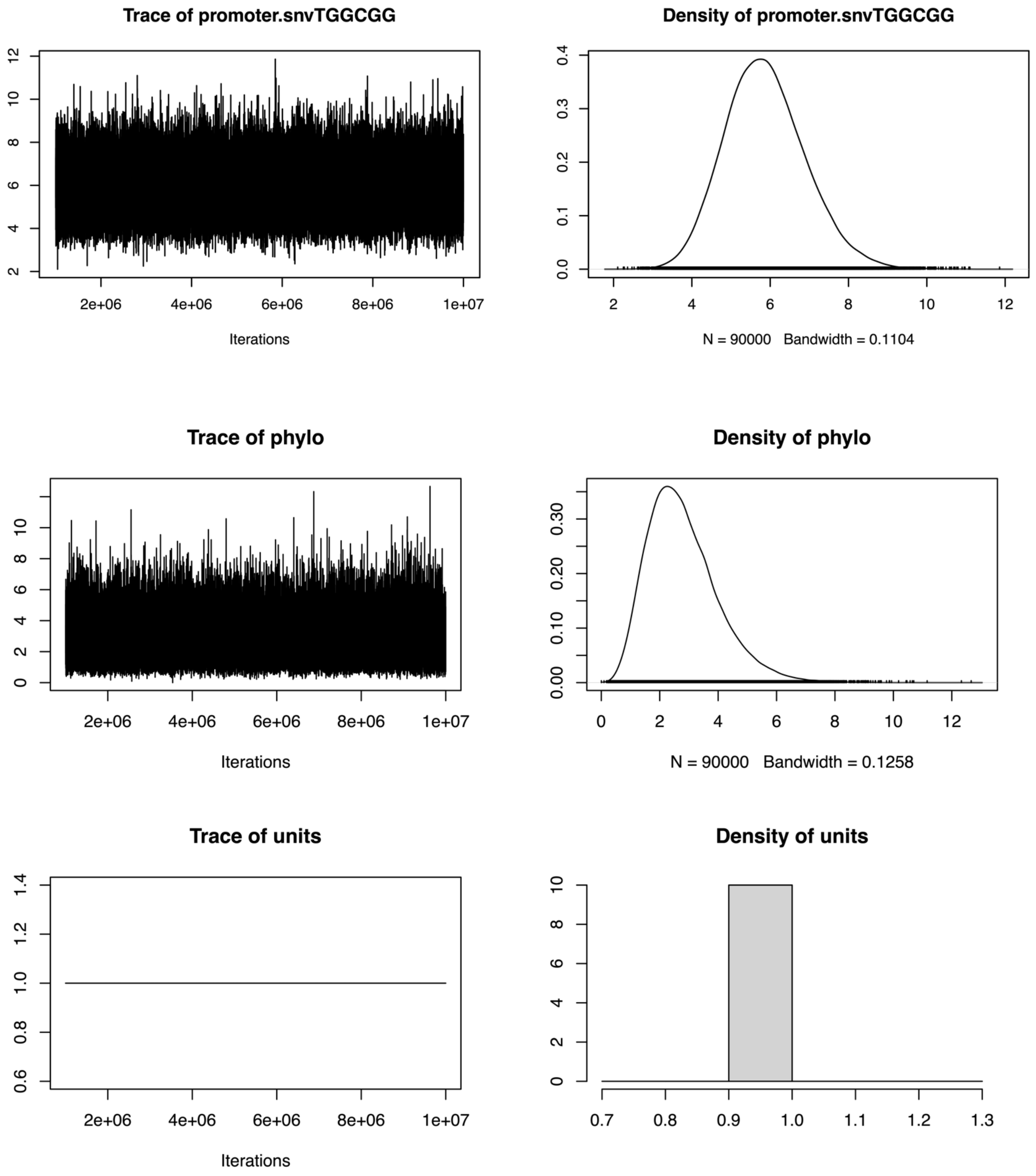


```
> mclist <- mcmc.list(chain.1$Sol, chain.2$Sol)
> gelman.diag(mclist)
Potential scale reduction factors:

                                Point est. Upper C.I.
(Intercept)                              1          1
tem1.isolate.copy.number.scaled          1          1
tem1.isolate.scaledTRUE                  1          1
ampc.promoter.snvAGCTTCTAGGG             1          1
```

ampc.promoter.snvAGCTCCTAGGG	1	1
ampc.promoter.snvGATTCCTAGGG	1	1
promoter.snvCGGCGA	1	1
promoter.snvTGGCGA	1	1
promoter.snvTGGCGG	1	1
acrfTRUE	1	1
phylo.542	1	1
phylo.416	1	1
phylo.56	1	1
phylo.213	1	1
phylo.54	1	1
phylo.435	1	1
phylo.90	1	1
phylo.393	1	1
phylo.72	1	1
phylo.217	1	1
phylo.489	1	1
phylo.189	1	1
phylo.434	1	1
phylo.194	1	1
phylo.371	1	1
phylo.127	1	1
phylo.99	1	1
phylo.129	1	1
phylo.8	1	1
phylo.414	1	1
phylo.454	1	1
phylo.259	1	1
phylo.120	1	1
phylo.145	1	1
phylo.426	1	1
phylo.46	1	1
phylo.49	1	1
phylo.394	1	1
phylo.183	1	1
phylo.30	1	1
phylo.151	1	1
phylo.148	1	1
phylo.236	1	1
phylo.220	1	1
phylo.117	1	1
phylo.272	1	1
phylo.400	1	1
phylo.328	1	1
phylo.415	1	1
phylo.398	1	1
phylo.252	1	1
phylo.239	1	1
phylo.246	1	1
phylo.98	1	1
phylo.424	1	1

phylo.66	1	1
phylo.108	1	1
phylo.476	1	1
phylo.468	1	1
phylo.363	1	1
phylo.21	1	1
phylo.128	1	1
phylo.257	1	1
phylo.242	1	1
phylo.339	1	1
phylo.455	1	1
phylo.149	1	1
phylo.417	1	1
phylo.332	1	1
phylo.373	1	1
phylo.402	1	1
phylo.52	1	1
phylo.498	1	1
phylo.494	1	1
phylo.472	1	1
phylo.302	1	1
phylo.445	1	1
phylo.389	1	1
phylo.61	1	1
phylo.299	1	1
phylo.420	1	1
phylo.103	1	1
phylo.464	1	1
phylo.449	1	1
phylo.97	1	1
phylo.208	1	1
phylo.329	1	1
phylo.316	1	1
phylo.80	1	1
phylo.438	1	1
phylo.216	1	1
phylo.324	1	1
phylo.224	1	1
phylo.447	1	1
phylo.419	1	1
phylo.175	1	1
phylo.266	1	1
phylo.343	1	1
phylo.136	1	1
phylo.156	1	1
phylo.144	1	1
phylo.404	1	1
phylo.202	1	1
phylo.439	1	1
phylo.366	1	1
phylo.285	1	1

phylo.297	1	1
phylo.133	1	1
phylo.284	1	1
phylo.512	1	1
phylo.155	1	1
phylo.253	1	1
phylo.37	1	1
phylo.317	1	1
phylo.143	1	1
phylo.475	1	1
phylo.342	1	1
phylo.471	1	1
phylo.264	1	1
phylo.517	1	1
phylo.507	1	1
phylo.132	1	1
phylo.39	1	1
phylo.286	1	1
phylo.170	1	1
phylo.76	1	1
phylo.500	1	1
phylo.524	1	1
phylo.533	1	1
phylo.333	1	1
phylo.410	1	1
phylo.446	1	1
phylo.344	1	1
phylo.406	1	1
phylo.433	1	1
phylo.22	1	1
phylo.430	1	1
phylo.288	1	1
phylo.270	1	1
phylo.511	1	1
phylo.429	1	1
phylo.277	1	1
phylo.322	1	1
phylo.482	1	1
phylo.262	1	1
phylo.387	1	1
phylo.58	1	1
phylo.193	1	1
phylo.388	1	1
phylo.158	1	1
phylo.12	1	1
phylo.110	1	1
phylo.276	1	1
phylo.412	1	1
phylo.411	1	1
phylo.365	1	1
phylo.82	1	1

phylo.92	1	1
phylo.492	1	1
phylo.370	1	1
phylo.137	1	1
phylo.483	1	1
phylo.267	1	1
phylo.421	1	1
phylo.237	1	1
phylo.200	1	1
phylo.536	1	1
phylo.260	1	1
phylo.537	1	1
phylo.477	1	1
phylo.518	1	1
phylo.522	1	1
phylo.55	1	1
phylo.358	1	1
phylo.197	1	1
phylo.493	1	1
phylo.41	1	1
phylo.40	1	1
phylo.34	1	1
phylo.94	1	1
phylo.408	1	1
phylo.292	1	1
phylo.248	1	1
phylo.109	1	1
phylo.1	1	1
phylo.214	1	1
phylo.480	1	1
phylo.423	1	1
phylo.247	1	1
phylo.268	1	1
phylo.157	1	1
phylo.385	1	1
phylo.353	1	1
phylo.53	1	1
phylo.211	1	1
phylo.265	1	1
phylo.139	1	1
phylo.198	1	1
phylo.396	1	1
phylo.456	1	1
phylo.245	1	1
phylo.263	1	1
phylo.134	1	1
phylo.3	1	1
phylo.95	1	1
phylo.514	1	1
phylo.525	1	1
phylo.529	1	1

phylo.162	1	1
phylo.515	1	1
phylo.282	1	1
phylo.427	1	1
phylo.240	1	1
phylo.166	1	1
phylo.60	1	1
phylo.182	1	1
phylo.532	1	1
phylo.273	1	1
phylo.119	1	1
phylo.11	1	1
phylo.355	1	1
phylo.241	1	1
phylo.315	1	1
phylo.24	1	1
phylo.203	1	1
phylo.121	1	1
phylo.305	1	1
phylo.534	1	1
phylo.167	1	1
phylo.336	1	1
phylo.16	1	1
phylo.330	1	1
phylo.320	1	1
phylo.188	1	1
phylo.274	1	1
phylo.280	1	1
phylo.281	1	1
phylo.89	1	1
phylo.531	1	1
phylo.106	1	1
phylo.227	1	1
phylo.164	1	1
phylo.382	1	1
phylo.192	1	1
phylo.301	1	1
phylo.506	1	1
phylo.334	1	1
phylo.177	1	1
phylo.469	1	1
phylo.2	1	1
phylo.470	1	1
phylo.351	1	1
phylo.31	1	1
phylo.159	1	1
phylo.187	1	1
phylo.488	1	1
phylo.486	1	1
phylo.225	1	1
phylo.32	1	1

phylo.541	1	1
phylo.380	1	1
phylo.462	1	1
phylo.451	1	1
phylo.403	1	1
phylo.115	1	1
phylo.250	1	1
phylo.93	1	1
phylo.230	1	1
phylo.457	1	1
phylo.313	1	1
phylo.331	1	1
phylo.176	1	1
phylo.75	1	1
phylo.296	1	1
phylo.205	1	1
phylo.256	1	1
phylo.88	1	1
phylo.487	1	1
phylo.347	1	1
phylo.442	1	1
phylo.495	1	1
phylo.116	1	1
phylo.233	1	1
phylo.539	1	1
phylo.83	1	1
phylo.444	1	1
phylo.508	1	1
phylo.33	1	1
phylo.57	1	1
phylo.142	1	1
phylo.311	1	1
phylo.386	1	1
phylo.135	1	1
phylo.19	1	1
phylo.59	1	1
phylo.287	1	1
phylo.375	1	1
phylo.490	1	1
phylo.448	1	1
phylo.10	1	1
phylo.399	1	1
phylo.466	1	1
phylo.310	1	1
phylo.543	1	1
phylo.504	1	1
phylo.174	1	1
phylo.499	1	1
phylo.126	1	1
phylo.413	1	1
phylo.125	1	1

phylo.201	1	1
phylo.513	1	1
phylo.69	1	1
phylo.340	1	1
phylo.179	1	1
phylo.25	1	1
phylo.436	1	1
phylo.546	1	1
phylo.279	1	1
phylo.485	1	1
phylo.38	1	1
phylo.422	1	1
phylo.229	1	1
phylo.35	1	1
phylo.131	1	1
phylo.71	1	1
phylo.6	1	1
phylo.28	1	1
phylo.401	1	1
phylo.222	1	1
phylo.243	1	1
phylo.42	1	1
phylo.63	1	1
phylo.18	1	1
phylo.123	1	1
phylo.140	1	1
phylo.502	1	1
phylo.503	1	1
phylo.341	1	1
phylo.238	1	1
phylo.478	1	1
phylo.443	1	1
phylo.44	1	1
phylo.544	1	1
phylo.204	1	1
phylo.510	1	1
phylo.314	1	1
phylo.124	1	1
phylo.64	1	1
phylo.219	1	1
phylo.178	1	1
phylo.345	1	1
phylo.479	1	1
phylo.307	1	1
phylo.275	1	1
phylo.540	1	1
phylo.23	1	1
phylo.73	1	1
phylo.530	1	1
phylo.244	1	1
phylo.191	1	1

```
phylo.474                                   1          1
phylo.460                                   1          1
phylo.528                                   1          1
phylo.295                                   1          1
phylo.459                                   1          1
phylo.463                                   1          1
phylo.79                                    1          1
phylo.481                                   1          1
phylo.190                                   1          1
phylo.461                                   1          1
phylo.535                                   1          1
phylo.223                                   1          1
phylo.29                                    1          1
phylo.65                                    1          1
phylo.283                                   1          1
phylo.425                                   1          1
phylo.255                                   1          1
phylo.521                                   1          1
phylo.199                                   1          1
phylo.107                                   1          1
phylo.294                                   1          1
phylo.122                                   1          1
phylo.87                                    1          1
phylo.105                                   1          1
phylo.138                                   1          1
phylo.440                                   1          1

Multivariate psrf

1
```

Combined phenotype model specification

The combined model investigates how *bla*$_{\text{TEM-1}}$ expression affects MIC, accounting for phylogenetic effects and scaling these effects by a parameter *θ*. The random effects structure for expression for isolate i is given by

$$\text{exp}_i = p_i + a_i$$

where p_i is the phylogenetic effect and a_i is the isolate main effect. The random effects structure for MIC for isolate i is given by

$$\text{MIC}_i = \theta(p_i + a_i) + u_i$$

where θ is the scaling factor and u_i phylogenetic effect specifically fitted for MIC.

The covariance structure can be represented as a series of regressions based on the random effects. The variance of phylogenetic effect on expression is given by

$$\text{Var}(p) = \sigma_p^2$$

the covariance between expression and MIC is given by

$$\mathrm{Cov}(exp, MIC) = \theta \times \sigma_p^2$$

and the variance of MIC is given by

$$\mathrm{Var}(MIC) = \mathrm{Var}(u) \ + \theta^2 \times \sigma_p^2$$

This approach demonstrates that the MIC phenotype is influenced by the expression levels, with the relationship modulated by the scaling parameter *θ*. The model assumes a causal relationship where expression impacts MIC, and this impact is consistent across all random effects. The combined model with fixed and random effects is then given by

$$Y_i = \begin{cases} X_i^{exp}\boldsymbol{\beta} + \mathrm{exp}_i \ + \ \varepsilon_i^{exp} & \text{for expression} \\ X_i^{MIC}\boldsymbol{\beta} + \mathrm{MIC}_i \ + \ \varepsilon_i^{MIC} & \text{for MIC} \end{cases}$$

Note, that due to model complexity, we used ordinal encoding for MIC categories.

Combined phenotype model outputs

```
> summary(chain.1)

 Iterations = 1000001:9999901
 Thinning interval  = 100
 Sample size  = 90000 

 DIC: NaN 

 G-structure:  ~phylo

      post.mean  l-95% CI u-95% CI eff.samp
phylo   0.09938 1.489e-08   0.2427    57787

               ~isolate.id

           post.mean l-95% CI u-95% CI eff.samp
isolate.id    0.1818   0.1039   0.2705    50740

               ~us(at.level(trait, "mic")):phylo

                                                              post.mean
l-95% CI u-95% CI eff.samp
at.level(trait, "mic"):at.level(trait, "mic").phylo    0.5906
0.1418      1.1    83991

 R-structure:  ~idh(trait):units

               post.mean l-95% CI u-95% CI eff.samp
```

```
traitexp.units   0.04027  0.03245  0.04857    15534
traitmic.units   0.41560  0.23881  0.60102    57755

 Location effects: y ~ -1 + trait:(1 + tem1.isolate.scaled + 
tem1.isolate.copy.number.scaled + promoter.snv) 

                                         post.mean l-95% CI u-
95% CI eff.samp  pMCMC    
(Intercept)                                4.32938  3.87446  
4.78219    90000 <1e-05 ***
traitexp:tem1.isolate.scaledFALSE         -4.54617 -5.19684 -
3.92338    90000 <1e-05 ***
traitmic:tem1.isolate.scaledFALSE         -0.46167 -0.91183 -
0.00569    90000 0.0465 *  
traitexp:tem1.isolate.copy.number.scaled  -1.02103 -1.96612 -
0.09322    88080 0.0349 *  
traitmic:tem1.isolate.copy.number.scaled   1.03191  0.72891  
1.34028    88658 <1e-05 ***
traitexp:promoter.snvCGGCGA               -0.24776 -0.50319  
0.01327    86917 0.0623 .  
traitmic:promoter.snvCGGCGA                0.03181 -0.23004  
0.28456    90000 0.8064    
traitexp:promoter.snvTGGCGA               -1.66270 -2.01689 -
1.30631    87829 <1e-05 ***
traitmic:promoter.snvTGGCGA                2.35052  1.83735  
2.88213    90000 <1e-05 ***
traitexp:promoter.snvTGGCGG               -1.68334 -2.06755 -
1.30517    87857 <1e-05 ***
traitmic:promoter.snvTGGCGG                2.23015  1.69807  
2.77481    90000 <1e-05 ***
---
Signif. codes:  0 '***' 0.001 '**' 0.01 '*' 0.05 '.' 0.1 ' ' 1

 Theta scale parameter:

            post.mean l-95% CI u-95% CI eff.samp   pMCMC   
theta_scale   -1.1267  -1.4853  -0.7196     8506 0.00231 **
---
Signif. codes:  0 '***' 0.001 '**' 0.01 '*' 0.05 '.' 0.1 ' ' 1

> autocorr.diag(chain.1$VCV)
               phylo   isolate.id at.level(trait, 
"mic"):at.level(trait, "mic").phylo traitexp.units 
traitmic.units
Lag 0    1.000000000  1.000000000                                 
1.000000e+00   1.0000000000   1.0000000000
Lag 100  0.112367643  0.072780947                                 
1.409324e-02   0.2537680979   0.0829974214
Lag 500  0.015074121  0.022672690                                 
6.601437e-03   0.1825237734   0.0196155673
```

```
Lag 1000 0.006205217  0.009637130
-1.172422e-03   0.1062380205   0.0085013712
Lag 5000 0.002281869 -0.001722614
5.754047e-05   0.0001475553  -0.0005311794

> plot(chain.1)
```

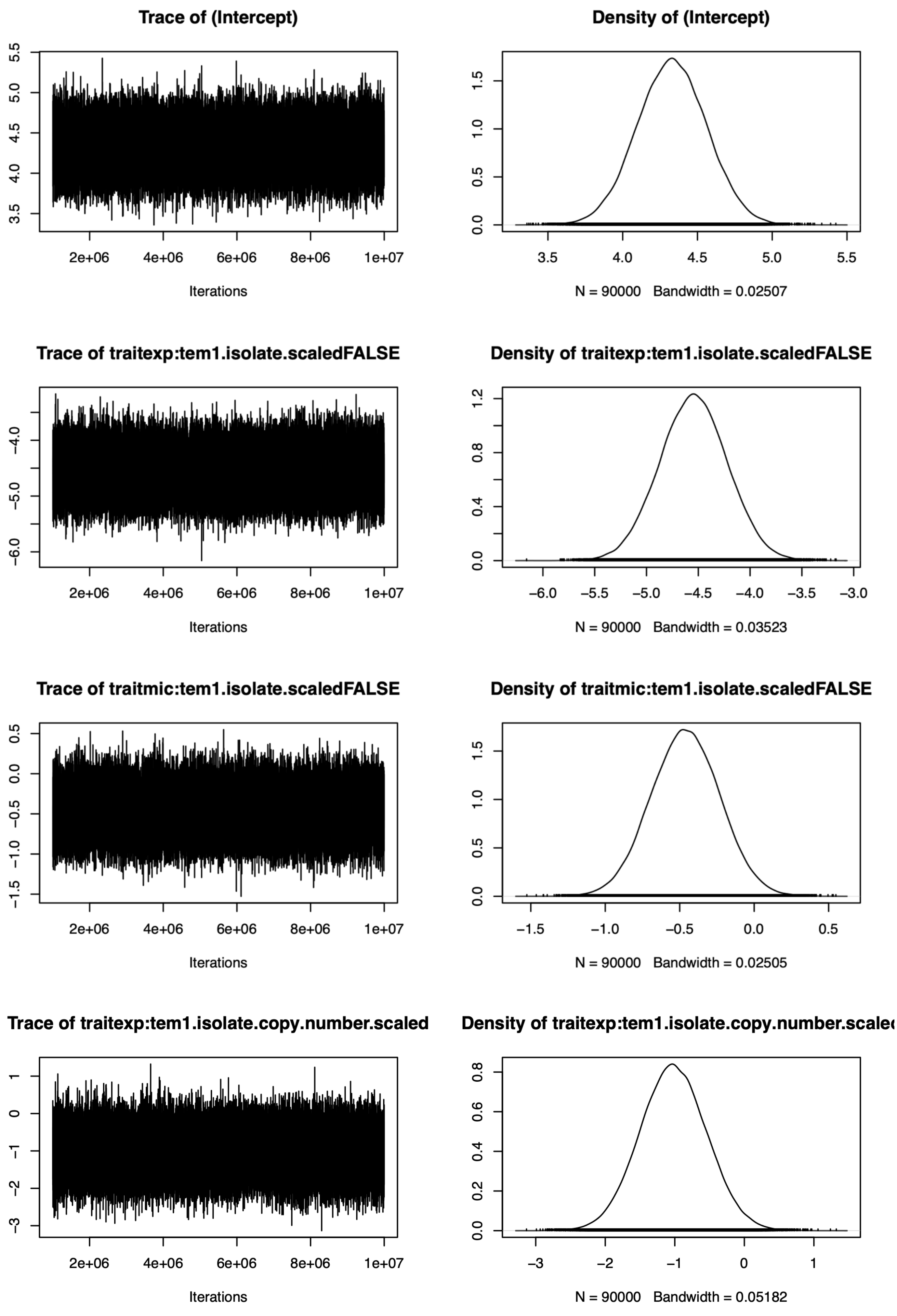

Trace of (Intercept)
Iterations
Density of (Intercept)
N = 90000 Bandwidth = 0.02507
Trace of traitexp:tem1.isolate.scaledFALSE
Iterations
Density of traitexp:tem1.isolate.scaledFALSE
N = 90000 Bandwidth = 0.03523
Trace of traitmic:tem1.isolate.scaledFALSE
Iterations
Density of traitmic:tem1.isolate.scaledFALSE
N = 90000 Bandwidth = 0.02505
Trace of traitexp:tem1.isolate.copy.number.scaled
Iterations
Density of traitexp:tem1.isolate.copy.number.scale
N = 90000 Bandwidth = 0.05182

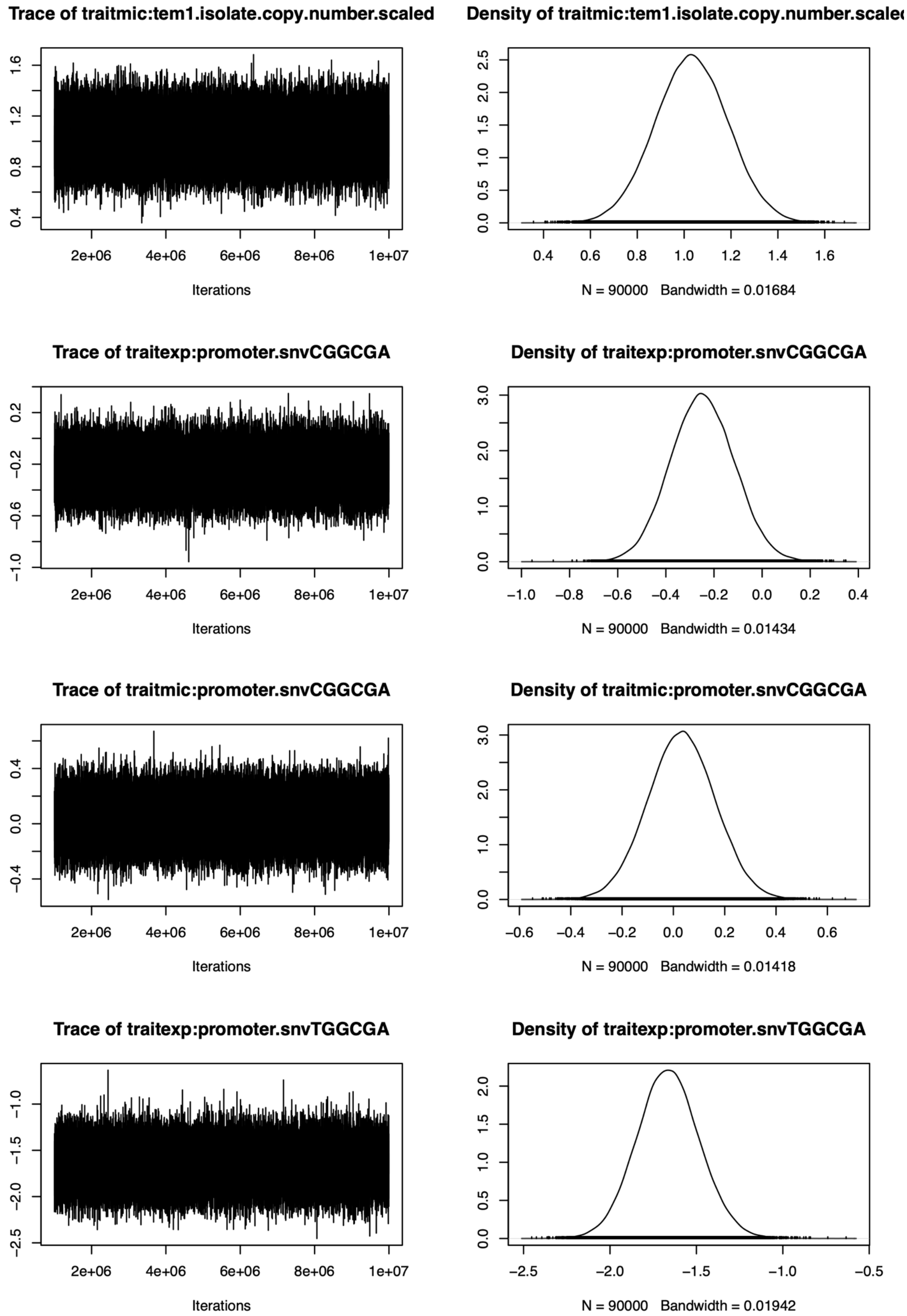
Trace of traitmic:tem1.isolate.copy.number.scaled
Iterations
Density of traitmic:tem1.isolate.copy.number.scale
N = 90000 Bandwidth = 0.01684
Trace of traitexp:promoter.snvCGGCGA
Iterations
Density of traitexp:promoter.snvCGGCGA
N = 90000 Bandwidth = 0.01434
Trace of traitmic:promoter.snvCGGCGA
Iterations
Density of traitmic:promoter.snvCGGCGA
N = 90000 Bandwidth = 0.01418
Trace of traitexp:promoter.snvTGGCGA
Iterations
Density of traitexp:promoter.snvTGGCGA
N = 90000 Bandwidth = 0.01942

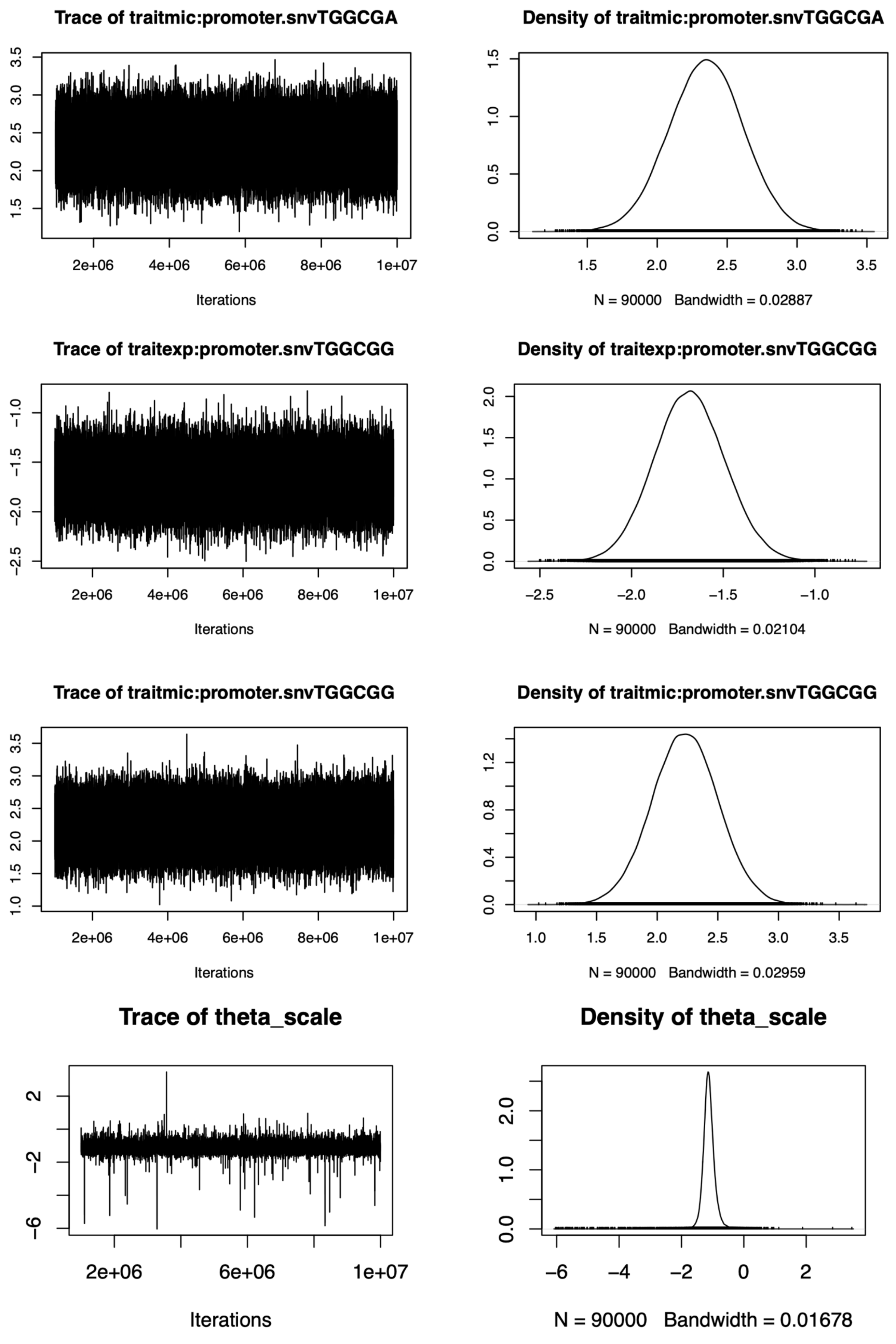

Trace of traitmic:promoter.snvTGGCGA
Iterations
Density of traitmic:promoter.snvTGGCGA
N = 90000 Bandwidth = 0.02887
Trace of traitexp:promoter.snvTGGCGG
Iterations
Density of traitexp:promoter.snvTGGCGG
N = 90000 Bandwidth = 0.02104
Trace of traitmic:promoter.snvTGGCGG
Iterations
Density of traitmic:promoter.snvTGGCGG
N = 90000 Bandwidth = 0.02959
Trace of theta_scale
Iterations
Density of theta_scale
N = 90000 Bandwidth = 0.01678

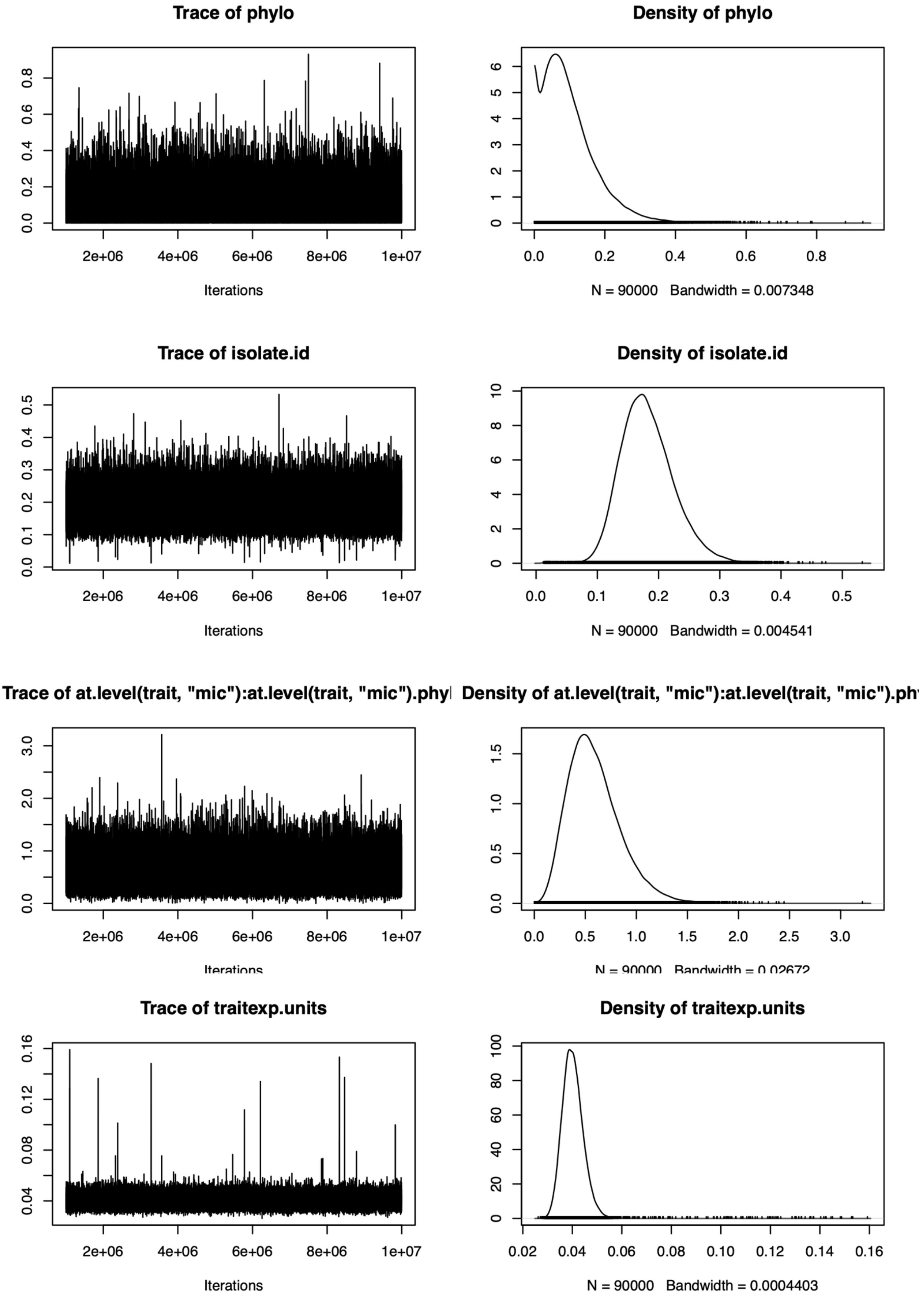
Trace of phylo
Iterations
Density of phylo
N = 90000 Bandwidth = 0.007348
Trace of isolate.id
Iterations
Density of isolate.id
N = 90000 Bandwidth = 0.004541
Trace of at.level(trait, "mic"):at.level(trait, "mic").phyl
Iterations
Density of at.level(trait, "mic"):at.level(trait, "mic").phy
N = 90000 Bandwidth = 0.02672
Trace of traitexp.units
Iterations
Density of traitexp.units
N = 90000 Bandwidth = 0.0004403

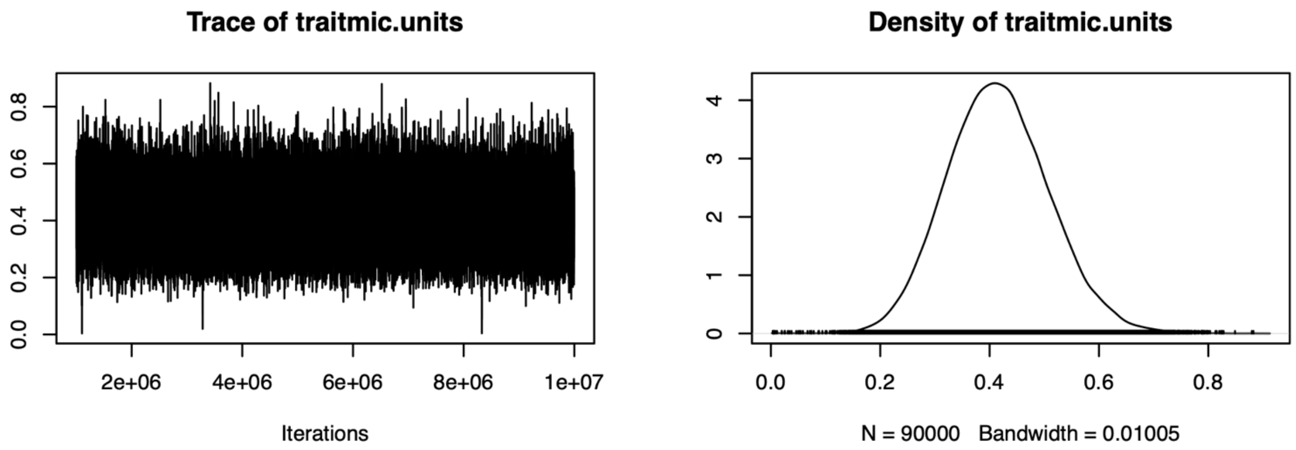


```
> summary(chain.2)

 Iterations = 1000001:9999901
 Thinning interval  = 100
 Sample size  = 90000

 DIC: NaN

 G-structure:  ~phylo

      post.mean  l-95% CI u-95% CI eff.samp
phylo    0.1003 6.355e-11   0.2435    61115

               ~isolate.id

           post.mean l-95% CI u-95% CI eff.samp
isolate.id    0.1818   0.1024   0.2703    48151

               ~us(at.level(trait, "mic")):phylo

                                                         post.mean
l-95% CI u-95% CI eff.samp
at.level(trait, "mic"):at.level(trait, "mic").phylo    0.5897
0.1465    1.095    84462

 R-structure:  ~idh(trait):units

               post.mean l-95% CI u-95% CI eff.samp
traitexp.units   0.04026  0.03233   0.0484    13141
traitmic.units   0.41494  0.23108   0.5954    56352

 Location effects: y ~ -1 + trait:(1 + tem1.isolate.scaled +
tem1.isolate.copy.number.scaled + promoter.snv)

                                            post.mean  l-95% CI
u-95% CI eff.samp  pMCMC
(Intercept)                                  4.328525  3.874485
4.782199    90000 <1e-05 ***
traitexp:tem1.isolate.scaledFALSE          -4.544511 -5.171154 -
3.902730    90000 <1e-05 ***
```

```
traitmic:tem1.isolate.scaledFALSE        -0.461352 -0.899921
0.002839    90000 0.0448 *
traitexp:tem1.isolate.copy.number.scaled -1.019478 -1.946873 -
0.062113    90000 0.0352 *
traitmic:tem1.isolate.copy.number.scaled  1.033209  0.729652
1.336409    90092 <1e-05 ***
traitexp:promoter.snvCGGCGA              -0.248080 -0.507897
0.008901    90000 0.0627 .
traitmic:promoter.snvCGGCGA               0.032095 -0.223837
0.290353    90000 0.8044
traitexp:promoter.snvTGGCGA              -1.662544 -2.014827 -
1.300562    83716 <1e-05 ***
traitmic:promoter.snvTGGCGA               2.351595  1.831656
2.880991    91212 <1e-05 ***
traitexp:promoter.snvTGGCGG              -1.683006 -2.056777 -
1.290550    87655 <1e-05 ***
traitmic:promoter.snvTGGCGG               2.230071  1.706237
2.771809    90000 <1e-05 ***
---
Signif. codes:  0 '***' 0.001 '**' 0.01 '*' 0.05 '.' 0.1 ' ' 1

 Theta scale parameter:

            post.mean l-95% CI u-95% CI eff.samp   pMCMC
theta_scale   -1.1272  -1.4934  -0.7211     8575 0.00198 **
---
Signif. codes:  0 '***' 0.001 '**' 0.01 '*' 0.05 '.' 0.1 ' ' 1

> autocorr.diag(chain.2$VCV)
                phylo   isolate.id at.level(trait,
"mic"):at.level(trait, "mic").phylo traitexp.units
traitmic.units
Lag 0    1.0000000000  1.000000000
1.000000000    1.000000000    1.000000000
Lag 100  0.1147704162  0.076378596
0.008282837    0.307716514    0.086258143
Lag 500  0.0125540987  0.032054763
-0.002772776    0.191916176    0.023457348
Lag 1000 0.0003901405  0.015048908
0.007131786    0.120163028    0.014677994
Lag 5000 0.0022367820 -0.001287983
0.003862192   -0.001317448    0.001987818

> plot(chain.2)
```

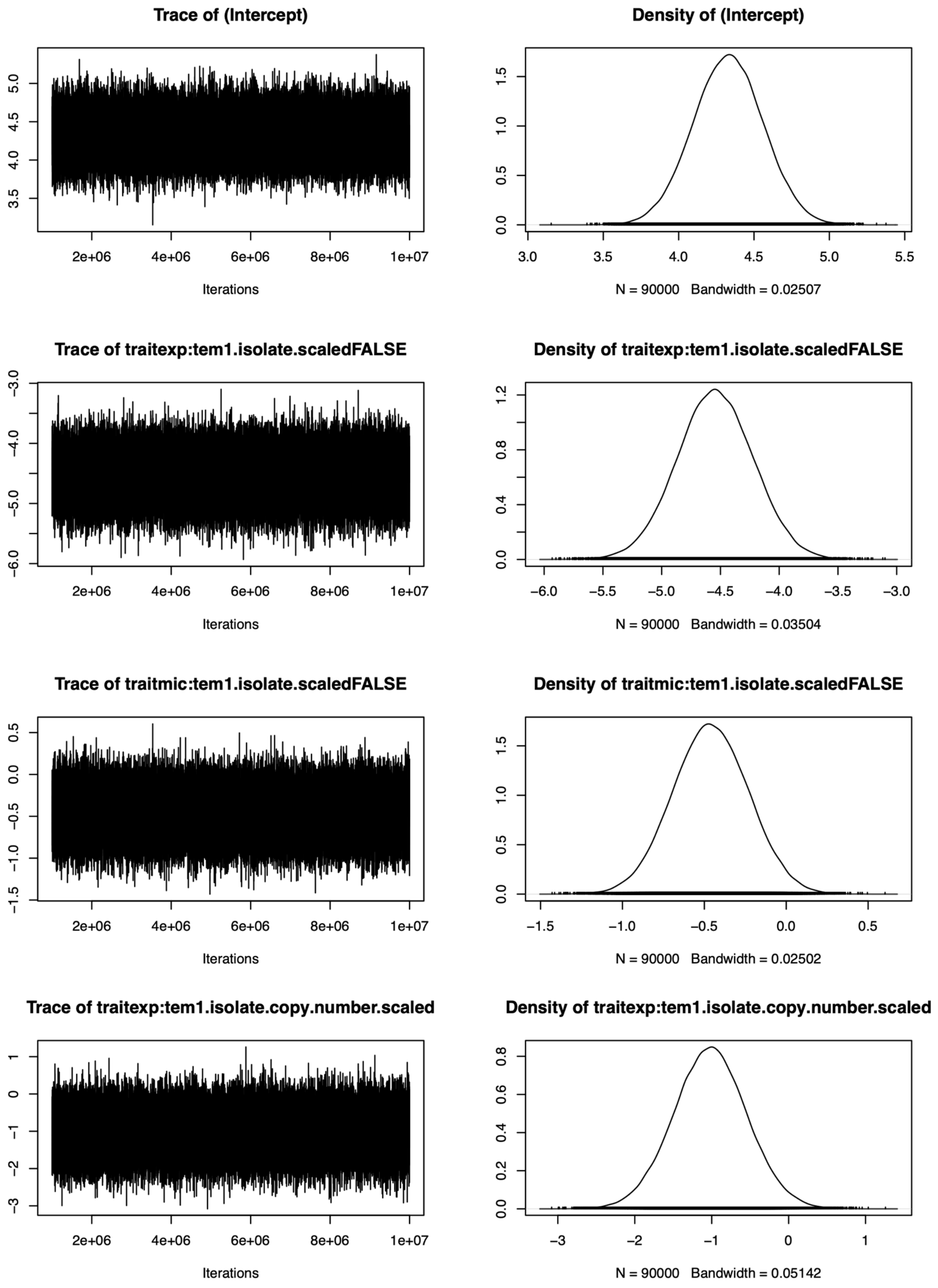

Trace of (Intercept)
Iterations
Density of (Intercept)
N = 90000 Bandwidth = 0.02507
Trace of traitexp:tem1.isolate.scaledFALSE
Iterations
Density of traitexp:tem1.isolate.scaledFALSE
N = 90000 Bandwidth = 0.03504
Trace of traitmic:tem1.isolate.scaledFALSE
Iterations
Density of traitmic:tem1.isolate.scaledFALSE
N = 90000 Bandwidth = 0.02502
Trace of traitexp:tem1.isolate.copy.number.scaled
Iterations
Density of traitexp:tem1.isolate.copy.number.scaled
N = 90000 Bandwidth = 0.05142

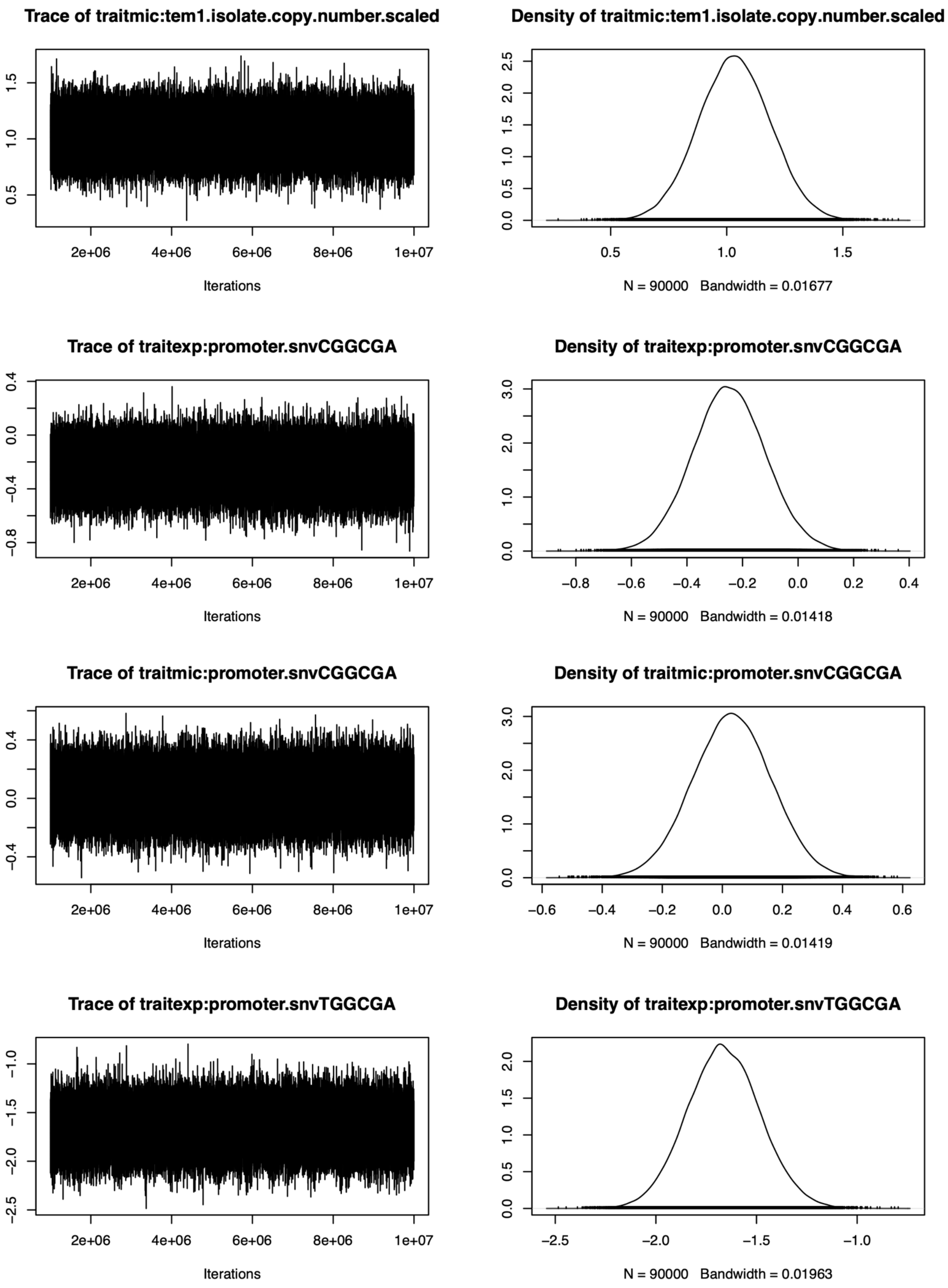

Trace of traitmic:tem1.isolate.copy.number.scaled
0.5
1.0
1.5
2e+06
4e+06
6e+06
8e+06
1e+07
Iterations
Density of traitmic:tem1.isolate.copy.number.scaled
0.0
0.5
1.0
1.5
2.0
2.5
0.5
1.0
1.5
N = 90000 Bandwidth = 0.01677
Trace of traitexp:promoter.snvCGGCGA
−0.8
−0.4
0.0
0.4
2e+06
4e+06
6e+06
8e+06
1e+07
Iterations
Density of traitexp:promoter.snvCGGCGA
0.0
1.0
2.0
3.0
−0.8
−0.6
−0.4
−0.2
0.0
0.2
0.4
N = 90000 Bandwidth = 0.01418
Trace of traitmic:promoter.snvCGGCGA
−0.4
0.0
0.4
2e+06
4e+06
6e+06
8e+06
1e+07
Iterations
Density of traitmic:promoter.snvCGGCGA
0.0
1.0
2.0
3.0
−0.6
−0.4
−0.2
0.0
0.2
0.4
0.6
N = 90000 Bandwidth = 0.01419
Trace of traitexp:promoter.snvTGGCGA
−2.5
−2.0
−1.5
−1.0
2e+06
4e+06
6e+06
8e+06
1e+07
Iterations
Density of traitexp:promoter.snvTGGCGA
0.0
0.5
1.0
1.5
2.0
−2.5
−2.0
−1.5
−1.0
N = 90000 Bandwidth = 0.01963

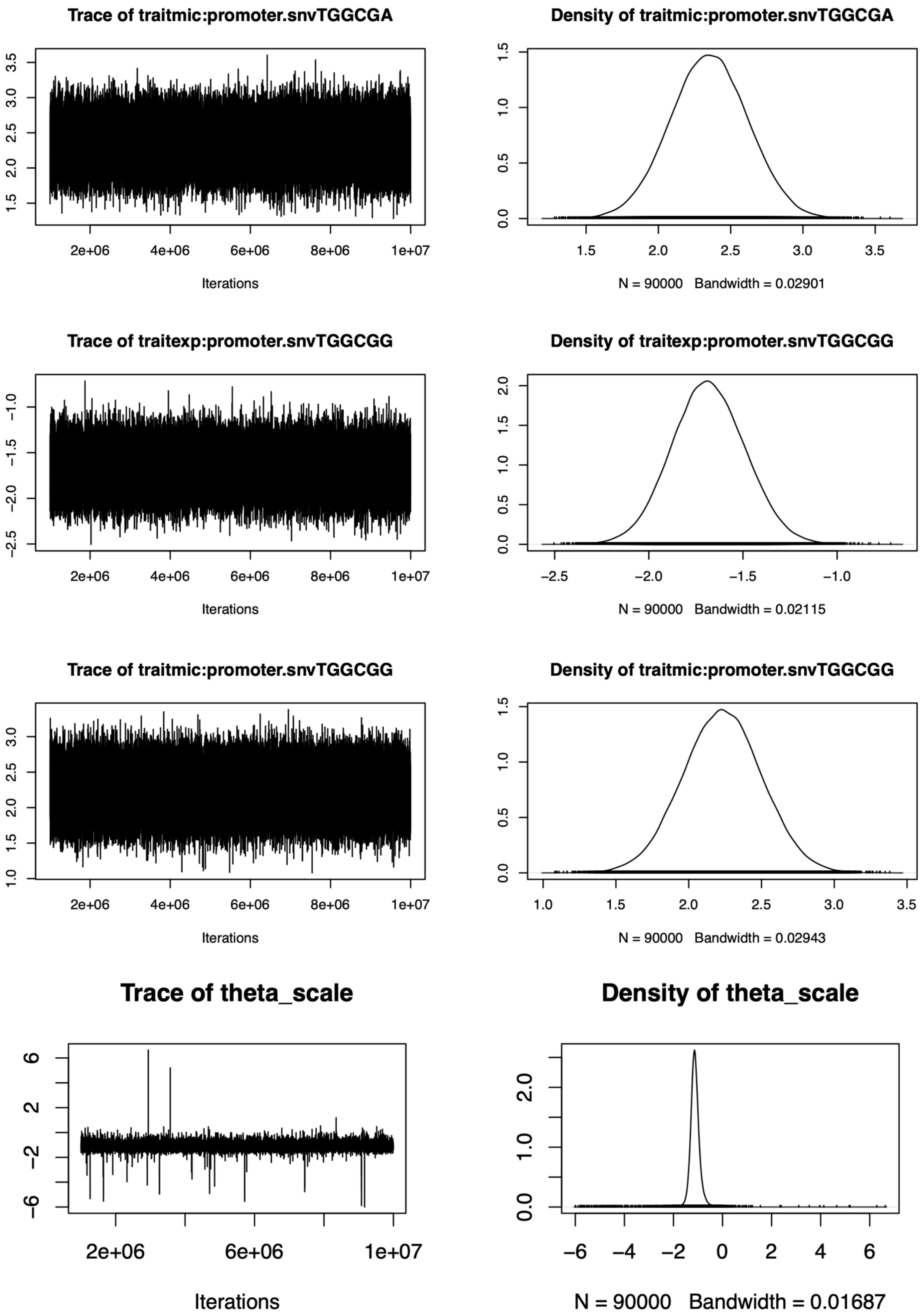
Trace of traitmic:promoter.snvTGGCGA
Iterations
Density of traitmic:promoter.snvTGGCGA
N = 90000 Bandwidth = 0.02901
Trace of traitexp:promoter.snvTGGCGG
Iterations
Density of traitexp:promoter.snvTGGCGG
N = 90000 Bandwidth = 0.02115
Trace of traitmic:promoter.snvTGGCGG
Iterations
Density of traitmic:promoter.snvTGGCGG
N = 90000 Bandwidth = 0.02943
Trace of theta_scale
Iterations
Density of theta_scale
N = 90000 Bandwidth = 0.01687

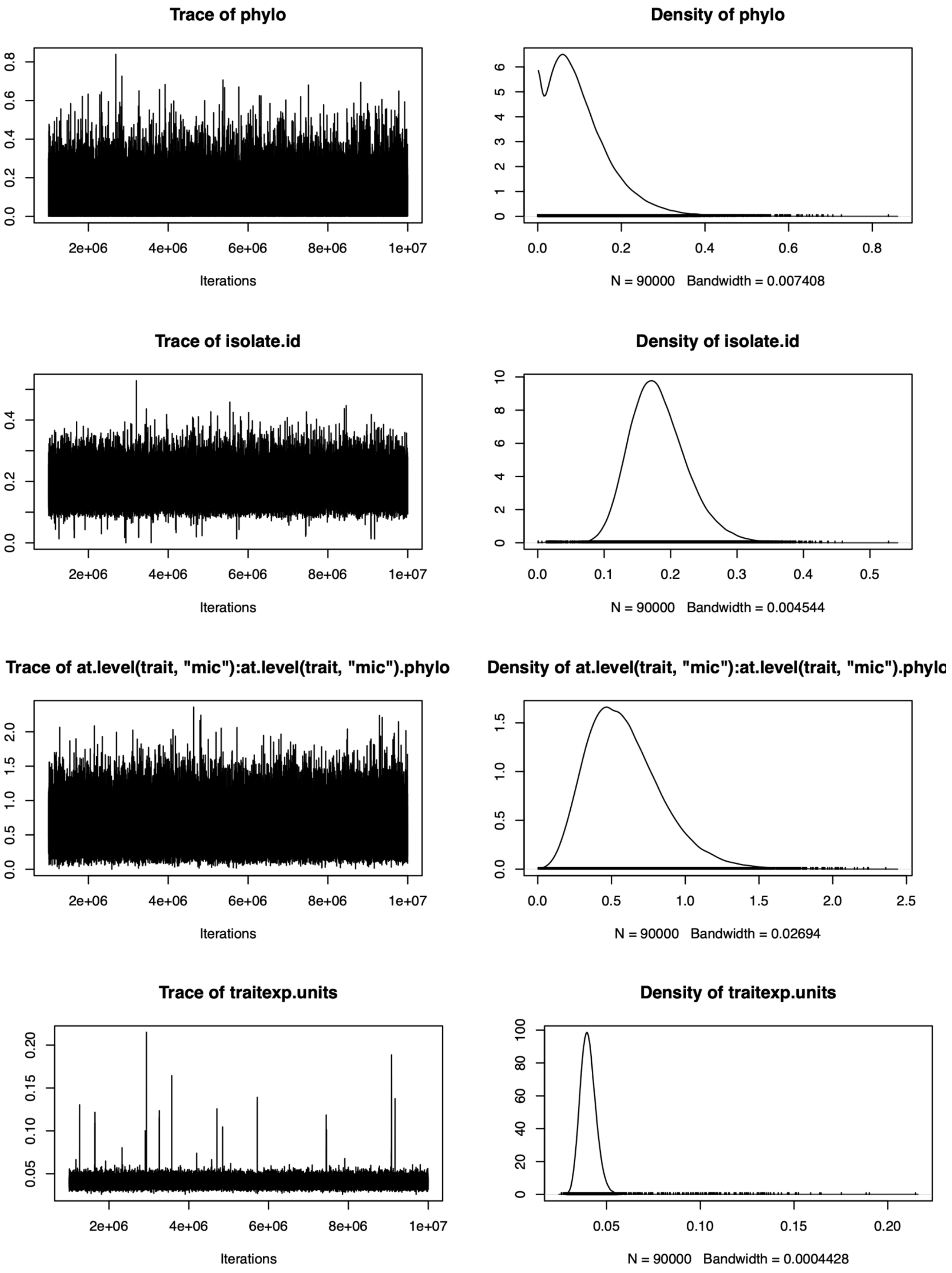
Trace of phylo
Iterations
Density of phylo
N = 90000 Bandwidth = 0.007408
Trace of isolate.id
Iterations
Density of isolate.id
N = 90000 Bandwidth = 0.004544
Trace of at.level(trait, "mic"):at.level(trait, "mic").phylo
Iterations
Density of at.level(trait, "mic"):at.level(trait, "mic").phylo
N = 90000 Bandwidth = 0.02694
Trace of traitexp.units
Iterations
Density of traitexp.units
N = 90000 Bandwidth = 0.0004428

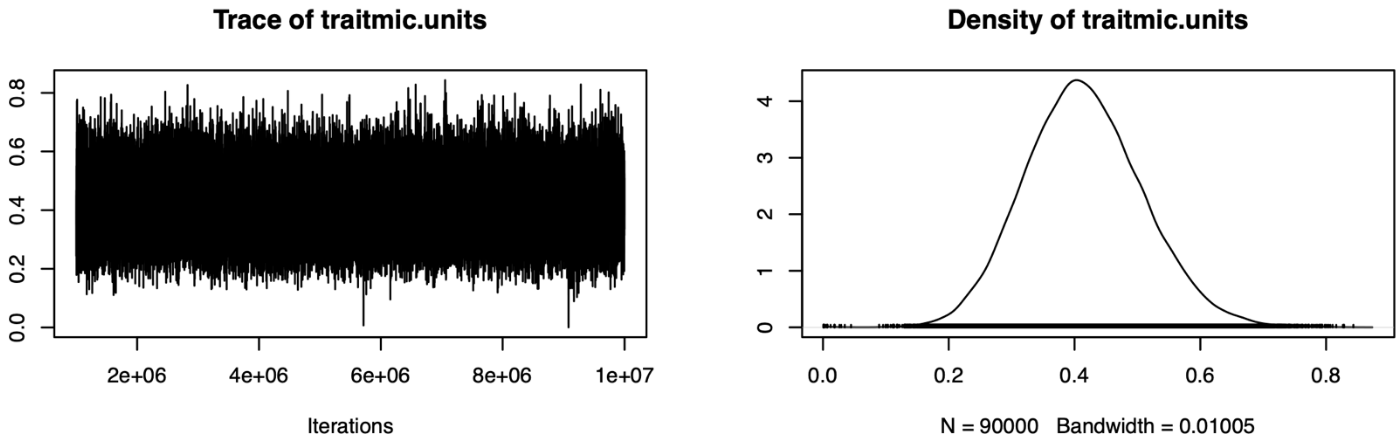


```
> mclist <- mcmc.list(chain.1$Sol, chain.2$Sol)
> gelman.diag(mclist)

Potential scale reduction factors:

                                          Point est. Upper C.I.
(Intercept)                                        1          1
traitexp:tem1.isolate.scaledFALSE                  1          1
traitmic:tem1.isolate.scaledFALSE                  1          1
traitexp:tem1.isolate.copy.number.scaled           1          1
traitmic:tem1.isolate.copy.number.scaled           1          1
traitexp:promoter.snvCGGCGA                        1          1
traitmic:promoter.snvCGGCGA                        1          1
traitexp:promoter.snvTGGCGA                        1          1
traitmic:promoter.snvTGGCGA                        1          1
traitexp:promoter.snvTGGCGG                        1          1
traitmic:promoter.snvTGGCGG                        1          1
phylo.542                                          1          1
phylo.416                                          1          1
phylo.56                                           1          1
phylo.213                                          1          1
phylo.54                                           1          1
phylo.435                                          1          1
phylo.90                                           1          1
phylo.393                                          1          1
phylo.72                                           1          1
phylo.489                                          1          1
phylo.189                                          1          1
phylo.434                                          1          1
phylo.194                                          1          1
phylo.371                                          1          1
phylo.127                                          1          1
phylo.99                                           1          1
phylo.129                                          1          1
phylo.8                                            1          1
phylo.414                                          1          1
phylo.454                                          1          1
phylo.120                                          1          1
phylo.145                                          1          1
phylo.426                                          1          1
```

phylo.394	1	1
phylo.183	1	1
phylo.30	1	1
phylo.151	1	1
phylo.148	1	1
phylo.236	1	1
phylo.220	1	1
phylo.117	1	1
phylo.272	1	1
phylo.400	1	1
phylo.328	1	1
phylo.415	1	1
phylo.398	1	1
phylo.252	1	1
phylo.246	1	1
phylo.424	1	1
phylo.476	1	1
phylo.468	1	1
phylo.363	1	1
phylo.21	1	1
phylo.128	1	1
phylo.257	1	1
phylo.242	1	1
phylo.339	1	1
phylo.149	1	1
phylo.445	1	1
phylo.61	1	1
phylo.299	1	1
phylo.420	1	1
phylo.103	1	1
phylo.464	1	1
phylo.449	1	1
phylo.97	1	1
phylo.208	1	1
phylo.329	1	1
phylo.316	1	1
phylo.80	1	1
phylo.438	1	1
phylo.216	1	1
phylo.324	1	1
phylo.224	1	1
phylo.447	1	1
phylo.266	1	1
phylo.136	1	1
phylo.156	1	1
phylo.144	1	1
phylo.404	1	1
phylo.202	1	1
phylo.439	1	1
phylo.366	1	1
phylo.285	1	1

phylo.133	1	1
phylo.284	1	1
phylo.512	1	1
phylo.155	1	1
phylo.37	1	1
phylo.317	1	1
phylo.143	1	1
phylo.475	1	1
phylo.342	1	1
phylo.471	1	1
phylo.264	1	1
phylo.517	1	1
phylo.507	1	1
phylo.132	1	1
phylo.39	1	1
phylo.286	1	1
phylo.524	1	1
phylo.533	1	1
phylo.333	1	1
phylo.410	1	1
phylo.446	1	1
phylo.344	1	1
phylo.406	1	1
phylo.22	1	1
phylo.430	1	1
phylo.288	1	1
phylo.511	1	1
phylo.429	1	1
phylo.277	1	1
phylo.322	1	1
phylo.262	1	1
phylo.387	1	1
phylo.58	1	1
phylo.388	1	1
phylo.158	1	1
phylo.12	1	1
phylo.276	1	1
phylo.412	1	1
phylo.411	1	1
phylo.365	1	1
phylo.82	1	1
phylo.92	1	1
phylo.370	1	1
phylo.137	1	1
phylo.267	1	1
phylo.421	1	1
phylo.237	1	1
phylo.200	1	1
phylo.536	1	1
phylo.260	1	1
phylo.537	1	1

phylo.477	1	1
phylo.518	1	1
phylo.55	1	1
phylo.358	1	1
phylo.197	1	1
phylo.493	1	1
phylo.41	1	1
phylo.40	1	1
phylo.408	1	1
phylo.292	1	1
phylo.248	1	1
phylo.1	1	1
phylo.214	1	1
phylo.480	1	1
phylo.423	1	1
phylo.247	1	1
phylo.268	1	1
phylo.353	1	1
phylo.211	1	1
phylo.265	1	1
phylo.139	1	1
phylo.198	1	1
phylo.396	1	1
phylo.263	1	1
phylo.95	1	1
phylo.162	1	1
phylo.515	1	1
phylo.282	1	1
phylo.427	1	1
phylo.240	1	1
phylo.166	1	1
phylo.182	1	1
phylo.532	1	1
phylo.273	1	1
phylo.119	1	1
phylo.355	1	1
phylo.241	1	1
phylo.203	1	1
phylo.121	1	1
phylo.305	1	1
phylo.534	1	1
phylo.167	1	1
phylo.336	1	1
phylo.188	1	1
phylo.274	1	1
phylo.281	1	1
phylo.89	1	1
phylo.531	1	1
phylo.164	1	1
phylo.382	1	1
phylo.192	1	1

phylo.301	1	1
phylo.506	1	1
phylo.334	1	1
phylo.469	1	1
phylo.2	1	1
phylo.470	1	1
phylo.351	1	1
phylo.31	1	1
phylo.159	1	1
phylo.187	1	1
phylo.488	1	1
phylo.486	1	1
phylo.225	1	1
phylo.32	1	1
phylo.541	1	1
phylo.380	1	1
phylo.462	1	1
phylo.451	1	1
phylo.403	1	1
phylo.115	1	1
phylo.250	1	1
phylo.93	1	1
phylo.230	1	1
phylo.457	1	1
phylo.313	1	1
phylo.331	1	1
phylo.176	1	1
phylo.75	1	1
phylo.296	1	1
phylo.205	1	1
phylo.256	1	1
phylo.487	1	1
phylo.347	1	1
phylo.442	1	1
phylo.495	1	1
phylo.116	1	1
phylo.233	1	1
phylo.539	1	1
phylo.83	1	1
phylo.444	1	1
phylo.508	1	1
phylo.33	1	1
phylo.142	1	1
phylo.311	1	1
phylo.386	1	1
phylo.135	1	1
phylo.19	1	1
phylo.59	1	1
phylo.287	1	1
phylo.375	1	1
phylo.490	1	1

phylo.448	1	1
phylo.10	1	1
phylo.399	1	1
phylo.466	1	1
phylo.310	1	1
phylo.543	1	1
phylo.504	1	1
phylo.174	1	1
phylo.499	1	1
phylo.126	1	1
phylo.413	1	1
phylo.125	1	1
phylo.201	1	1
phylo.513	1	1
phylo.69	1	1
phylo.340	1	1
phylo.179	1	1
phylo.25	1	1
phylo.436	1	1
phylo.546	1	1
phylo.279	1	1
phylo.485	1	1
phylo.38	1	1
phylo.422	1	1
phylo.229	1	1
phylo.35	1	1
phylo.131	1	1
phylo.71	1	1
phylo.6	1	1
phylo.401	1	1
phylo.222	1	1
phylo.243	1	1
phylo.42	1	1
phylo.63	1	1
phylo.18	1	1
phylo.123	1	1
phylo.140	1	1
phylo.502	1	1
phylo.503	1	1
phylo.341	1	1
phylo.238	1	1
phylo.478	1	1
phylo.443	1	1
phylo.44	1	1
phylo.544	1	1
phylo.204	1	1
phylo.510	1	1
phylo.314	1	1
phylo.124	1	1
phylo.64	1	1
phylo.219	1	1

phylo.178	1	1
phylo.345	1	1
phylo.479	1	1
phylo.307	1	1
phylo.275	1	1
phylo.540	1	1
phylo.23	1	1
phylo.73	1	1
phylo.530	1	1
phylo.244	1	1
phylo.191	1	1
phylo.474	1	1
phylo.460	1	1
phylo.528	1	1
phylo.295	1	1
phylo.459	1	1
phylo.463	1	1
phylo.79	1	1
phylo.481	1	1
phylo.190	1	1
phylo.461	1	1
phylo.535	1	1
phylo.223	1	1
phylo.29	1	1
phylo.65	1	1
phylo.283	1	1
phylo.425	1	1
phylo.107	1	1
phylo.294	1	1
phylo.122	1	1
phylo.105	1	1
phylo.138	1	1
phylo.440	1	1
isolate.id.OXEC-1	1	1
isolate.id.OXEC-10	1	1
isolate.id.OXEC-103	1	1
isolate.id.OXEC-105	1	1
isolate.id.OXEC-107	1	1
isolate.id.OXEC-115	1	1
isolate.id.OXEC-116	1	1
isolate.id.OXEC-117	1	1
isolate.id.OXEC-119	1	1
isolate.id.OXEC-12	1	1
isolate.id.OXEC-120	1	1
isolate.id.OXEC-121	1	1
isolate.id.OXEC-122	1	1
isolate.id.OXEC-123	1	1
isolate.id.OXEC-124	1	1
isolate.id.OXEC-125	1	1
isolate.id.OXEC-126	1	1
isolate.id.OXEC-127	1	1

isolate.id.OXEC-128	1	1
isolate.id.OXEC-129	1	1
isolate.id.OXEC-131	1	1
isolate.id.OXEC-132	1	1
isolate.id.OXEC-133	1	1
isolate.id.OXEC-135	1	1
isolate.id.OXEC-136	1	1
isolate.id.OXEC-137	1	1
isolate.id.OXEC-138	1	1
isolate.id.OXEC-139	1	1
isolate.id.OXEC-140	1	1
isolate.id.OXEC-142	1	1
isolate.id.OXEC-143	1	1
isolate.id.OXEC-144	1	1
isolate.id.OXEC-145	1	1
isolate.id.OXEC-148	1	1
isolate.id.OXEC-149	1	1
isolate.id.OXEC-151	1	1
isolate.id.OXEC-155	1	1
isolate.id.OXEC-156	1	1
isolate.id.OXEC-158	1	1
isolate.id.OXEC-159	1	1
isolate.id.OXEC-162	1	1
isolate.id.OXEC-164	1	1
isolate.id.OXEC-166	1	1
isolate.id.OXEC-167	1	1
isolate.id.OXEC-174	1	1
isolate.id.OXEC-176	1	1
isolate.id.OXEC-178	1	1
isolate.id.OXEC-179	1	1
isolate.id.OXEC-18	1	1
isolate.id.OXEC-182	1	1
isolate.id.OXEC-183	1	1
isolate.id.OXEC-187	1	1
isolate.id.OXEC-188	1	1
isolate.id.OXEC-189	1	1
isolate.id.OXEC-19	1	1
isolate.id.OXEC-190	1	1
isolate.id.OXEC-191	1	1
isolate.id.OXEC-192	1	1
isolate.id.OXEC-194	1	1
isolate.id.OXEC-197	1	1
isolate.id.OXEC-198	1	1
isolate.id.OXEC-2	1	1
isolate.id.OXEC-200	1	1
isolate.id.OXEC-201	1	1
isolate.id.OXEC-202	1	1
isolate.id.OXEC-203	1	1
isolate.id.OXEC-204	1	1
isolate.id.OXEC-205	1	1
isolate.id.OXEC-208	1	1

isolate.id.OXEC-21	1	1
isolate.id.OXEC-211	1	1
isolate.id.OXEC-213	1	1
isolate.id.OXEC-214	1	1
isolate.id.OXEC-216	1	1
isolate.id.OXEC-219	1	1
isolate.id.OXEC-22	1	1
isolate.id.OXEC-220	1	1
isolate.id.OXEC-222	1	1
isolate.id.OXEC-223	1	1
isolate.id.OXEC-224	1	1
isolate.id.OXEC-225	1	1
isolate.id.OXEC-229	1	1
isolate.id.OXEC-23	1	1
isolate.id.OXEC-230	1	1
isolate.id.OXEC-233	1	1
isolate.id.OXEC-236	1	1
isolate.id.OXEC-237	1	1
isolate.id.OXEC-238	1	1
isolate.id.OXEC-240	1	1
isolate.id.OXEC-241	1	1
isolate.id.OXEC-242	1	1
isolate.id.OXEC-243	1	1
isolate.id.OXEC-244	1	1
isolate.id.OXEC-246	1	1
isolate.id.OXEC-247	1	1
isolate.id.OXEC-248	1	1
isolate.id.OXEC-25	1	1
isolate.id.OXEC-250	1	1
isolate.id.OXEC-252	1	1
isolate.id.OXEC-256	1	1
isolate.id.OXEC-257	1	1
isolate.id.OXEC-260	1	1
isolate.id.OXEC-262	1	1
isolate.id.OXEC-263	1	1
isolate.id.OXEC-264	1	1
isolate.id.OXEC-265	1	1
isolate.id.OXEC-266	1	1
isolate.id.OXEC-267	1	1
isolate.id.OXEC-268	1	1
isolate.id.OXEC-272	1	1
isolate.id.OXEC-273	1	1
isolate.id.OXEC-274	1	1
isolate.id.OXEC-275	1	1
isolate.id.OXEC-276	1	1
isolate.id.OXEC-277	1	1
isolate.id.OXEC-279	1	1
isolate.id.OXEC-281	1	1
isolate.id.OXEC-282	1	1
isolate.id.OXEC-283	1	1
isolate.id.OXEC-284	1	1

isolate.id.OXEC-285	1	1
isolate.id.OXEC-286	1	1
isolate.id.OXEC-287	1	1
isolate.id.OXEC-288	1	1
isolate.id.OXEC-29	1	1
isolate.id.OXEC-292	1	1
isolate.id.OXEC-294	1	1
isolate.id.OXEC-295	1	1
isolate.id.OXEC-296	1	1
isolate.id.OXEC-299	1	1
isolate.id.OXEC-30	1	1
isolate.id.OXEC-301	1	1
isolate.id.OXEC-305	1	1
isolate.id.OXEC-307	1	1
isolate.id.OXEC-31	1	1
isolate.id.OXEC-310	1	1
isolate.id.OXEC-311	1	1
isolate.id.OXEC-313	1	1
isolate.id.OXEC-314	1	1
isolate.id.OXEC-316	1	1
isolate.id.OXEC-317	1	1
isolate.id.OXEC-32	1	1
isolate.id.OXEC-322	1	1
isolate.id.OXEC-324	1	1
isolate.id.OXEC-328	1	1
isolate.id.OXEC-329	1	1
isolate.id.OXEC-33	1	1
isolate.id.OXEC-331	1	1
isolate.id.OXEC-333	1	1
isolate.id.OXEC-334	1	1
isolate.id.OXEC-336	1	1
isolate.id.OXEC-339	1	1
isolate.id.OXEC-340	1	1
isolate.id.OXEC-341	1	1
isolate.id.OXEC-342	1	1
isolate.id.OXEC-344	1	1
isolate.id.OXEC-345	1	1
isolate.id.OXEC-347	1	1
isolate.id.OXEC-35	1	1
isolate.id.OXEC-351	1	1
isolate.id.OXEC-353	1	1
isolate.id.OXEC-355	1	1
isolate.id.OXEC-358	1	1
isolate.id.OXEC-363	1	1
isolate.id.OXEC-365	1	1
isolate.id.OXEC-366	1	1
isolate.id.OXEC-37	1	1
isolate.id.OXEC-370	1	1
isolate.id.OXEC-371	1	1
isolate.id.OXEC-375	1	1
isolate.id.OXEC-38	1	1

isolate.id.OXEC-380	1	1
isolate.id.OXEC-382	1	1
isolate.id.OXEC-386	1	1
isolate.id.OXEC-387	1	1
isolate.id.OXEC-388	1	1
isolate.id.OXEC-39	1	1
isolate.id.OXEC-393	1	1
isolate.id.OXEC-394	1	1
isolate.id.OXEC-396	1	1
isolate.id.OXEC-398	1	1
isolate.id.OXEC-399	1	1
isolate.id.OXEC-40	1	1
isolate.id.OXEC-400	1	1
isolate.id.OXEC-401	1	1
isolate.id.OXEC-403	1	1
isolate.id.OXEC-404	1	1
isolate.id.OXEC-406	1	1
isolate.id.OXEC-408	1	1
isolate.id.OXEC-41	1	1
isolate.id.OXEC-410	1	1
isolate.id.OXEC-411	1	1
isolate.id.OXEC-412	1	1
isolate.id.OXEC-413	1	1
isolate.id.OXEC-414	1	1
isolate.id.OXEC-415	1	1
isolate.id.OXEC-416	1	1
isolate.id.OXEC-42	1	1
isolate.id.OXEC-420	1	1
isolate.id.OXEC-421	1	1
isolate.id.OXEC-422	1	1
isolate.id.OXEC-423	1	1
isolate.id.OXEC-424	1	1
isolate.id.OXEC-425	1	1
isolate.id.OXEC-426	1	1
isolate.id.OXEC-427	1	1
isolate.id.OXEC-429	1	1
isolate.id.OXEC-430	1	1
isolate.id.OXEC-434	1	1
isolate.id.OXEC-435	1	1
isolate.id.OXEC-436	1	1
isolate.id.OXEC-438	1	1
isolate.id.OXEC-439	1	1
isolate.id.OXEC-44	1	1
isolate.id.OXEC-440	1	1
isolate.id.OXEC-442	1	1
isolate.id.OXEC-443	1	1
isolate.id.OXEC-444	1	1
isolate.id.OXEC-445	1	1
isolate.id.OXEC-446	1	1
isolate.id.OXEC-447	1	1
isolate.id.OXEC-448	1	1

isolate.id.OXEC-449	1	1
isolate.id.OXEC-451	1	1
isolate.id.OXEC-454	1	1
isolate.id.OXEC-457	1	1
isolate.id.OXEC-459	1	1
isolate.id.OXEC-460	1	1
isolate.id.OXEC-461	1	1
isolate.id.OXEC-462	1	1
isolate.id.OXEC-463	1	1
isolate.id.OXEC-464	1	1
isolate.id.OXEC-466	1	1
isolate.id.OXEC-468	1	1
isolate.id.OXEC-469	1	1
isolate.id.OXEC-470	1	1
isolate.id.OXEC-471	1	1
isolate.id.OXEC-474	1	1
isolate.id.OXEC-475	1	1
isolate.id.OXEC-476	1	1
isolate.id.OXEC-477	1	1
isolate.id.OXEC-478	1	1
isolate.id.OXEC-479	1	1
isolate.id.OXEC-480	1	1
isolate.id.OXEC-481	1	1
isolate.id.OXEC-485	1	1
isolate.id.OXEC-486	1	1
isolate.id.OXEC-487	1	1
isolate.id.OXEC-488	1	1
isolate.id.OXEC-489	1	1
isolate.id.OXEC-490	1	1
isolate.id.OXEC-493	1	1
isolate.id.OXEC-495	1	1
isolate.id.OXEC-499	1	1
isolate.id.OXEC-502	1	1
isolate.id.OXEC-503	1	1
isolate.id.OXEC-504	1	1
isolate.id.OXEC-506	1	1
isolate.id.OXEC-507	1	1
isolate.id.OXEC-508	1	1
isolate.id.OXEC-510	1	1
isolate.id.OXEC-511	1	1
isolate.id.OXEC-512	1	1
isolate.id.OXEC-513	1	1
isolate.id.OXEC-515	1	1
isolate.id.OXEC-517	1	1
isolate.id.OXEC-518	1	1
isolate.id.OXEC-524	1	1
isolate.id.OXEC-528	1	1
isolate.id.OXEC-530	1	1
isolate.id.OXEC-531	1	1
isolate.id.OXEC-532	1	1
isolate.id.OXEC-533	1	1

isolate.id.OXEC-534	1	1
isolate.id.OXEC-535	1	1
isolate.id.OXEC-536	1	1
isolate.id.OXEC-537	1	1
isolate.id.OXEC-539	1	1
isolate.id.OXEC-54	1	1
isolate.id.OXEC-540	1	1
isolate.id.OXEC-541	1	1
isolate.id.OXEC-542	1	1
isolate.id.OXEC-543	1	1
isolate.id.OXEC-544	1	1
isolate.id.OXEC-546	1	1
isolate.id.OXEC-55	1	1
isolate.id.OXEC-56	1	1
isolate.id.OXEC-58	1	1
isolate.id.OXEC-59	1	1
isolate.id.OXEC-6	1	1
isolate.id.OXEC-61	1	1
isolate.id.OXEC-63	1	1
isolate.id.OXEC-64	1	1
isolate.id.OXEC-65	1	1
isolate.id.OXEC-69	1	1
isolate.id.OXEC-71	1	1
isolate.id.OXEC-72	1	1
isolate.id.OXEC-73	1	1
isolate.id.OXEC-75	1	1
isolate.id.OXEC-79	1	1
isolate.id.OXEC-8	1	1
isolate.id.OXEC-80	1	1
isolate.id.OXEC-82	1	1
isolate.id.OXEC-83	1	1
isolate.id.OXEC-89	1	1
isolate.id.OXEC-90	1	1
isolate.id.OXEC-92	1	1
isolate.id.OXEC-93	1	1
isolate.id.OXEC-95	1	1
isolate.id.OXEC-97	1	1
isolate.id.OXEC-99	1	1
at.level(trait, "mic").phylo.542	1	1
at.level(trait, "mic").phylo.416	1	1
at.level(trait, "mic").phylo.56	1	1
at.level(trait, "mic").phylo.213	1	1
at.level(trait, "mic").phylo.54	1	1
at.level(trait, "mic").phylo.435	1	1
at.level(trait, "mic").phylo.90	1	1
at.level(trait, "mic").phylo.393	1	1
at.level(trait, "mic").phylo.72	1	1
at.level(trait, "mic").phylo.489	1	1
at.level(trait, "mic").phylo.189	1	1
at.level(trait, "mic").phylo.434	1	1
at.level(trait, "mic").phylo.194	1	1

at.level(trait, "mic").phylo.371	1	1
at.level(trait, "mic").phylo.127	1	1
at.level(trait, "mic").phylo.99	1	1
at.level(trait, "mic").phylo.129	1	1
at.level(trait, "mic").phylo.8	1	1
at.level(trait, "mic").phylo.414	1	1
at.level(trait, "mic").phylo.454	1	1
at.level(trait, "mic").phylo.120	1	1
at.level(trait, "mic").phylo.145	1	1
at.level(trait, "mic").phylo.426	1	1
at.level(trait, "mic").phylo.394	1	1
at.level(trait, "mic").phylo.183	1	1
at.level(trait, "mic").phylo.30	1	1
at.level(trait, "mic").phylo.151	1	1
at.level(trait, "mic").phylo.148	1	1
at.level(trait, "mic").phylo.236	1	1
at.level(trait, "mic").phylo.220	1	1
at.level(trait, "mic").phylo.117	1	1
at.level(trait, "mic").phylo.272	1	1
at.level(trait, "mic").phylo.400	1	1
at.level(trait, "mic").phylo.328	1	1
at.level(trait, "mic").phylo.415	1	1
at.level(trait, "mic").phylo.398	1	1
at.level(trait, "mic").phylo.252	1	1
at.level(trait, "mic").phylo.246	1	1
at.level(trait, "mic").phylo.424	1	1
at.level(trait, "mic").phylo.476	1	1
at.level(trait, "mic").phylo.468	1	1
at.level(trait, "mic").phylo.363	1	1
at.level(trait, "mic").phylo.21	1	1
at.level(trait, "mic").phylo.128	1	1
at.level(trait, "mic").phylo.257	1	1
at.level(trait, "mic").phylo.242	1	1
at.level(trait, "mic").phylo.339	1	1
at.level(trait, "mic").phylo.149	1	1
at.level(trait, "mic").phylo.445	1	1
at.level(trait, "mic").phylo.61	1	1
at.level(trait, "mic").phylo.299	1	1
at.level(trait, "mic").phylo.420	1	1
at.level(trait, "mic").phylo.103	1	1
at.level(trait, "mic").phylo.464	1	1
at.level(trait, "mic").phylo.449	1	1
at.level(trait, "mic").phylo.97	1	1
at.level(trait, "mic").phylo.208	1	1
at.level(trait, "mic").phylo.329	1	1
at.level(trait, "mic").phylo.316	1	1
at.level(trait, "mic").phylo.80	1	1
at.level(trait, "mic").phylo.438	1	1
at.level(trait, "mic").phylo.216	1	1
at.level(trait, "mic").phylo.324	1	1
at.level(trait, "mic").phylo.224	1	1

at.level(trait, "mic").phylo.447	1	1
at.level(trait, "mic").phylo.266	1	1
at.level(trait, "mic").phylo.136	1	1
at.level(trait, "mic").phylo.156	1	1
at.level(trait, "mic").phylo.144	1	1
at.level(trait, "mic").phylo.404	1	1
at.level(trait, "mic").phylo.202	1	1
at.level(trait, "mic").phylo.439	1	1
at.level(trait, "mic").phylo.366	1	1
at.level(trait, "mic").phylo.285	1	1
at.level(trait, "mic").phylo.133	1	1
at.level(trait, "mic").phylo.284	1	1
at.level(trait, "mic").phylo.512	1	1
at.level(trait, "mic").phylo.155	1	1
at.level(trait, "mic").phylo.37	1	1
at.level(trait, "mic").phylo.317	1	1
at.level(trait, "mic").phylo.143	1	1
at.level(trait, "mic").phylo.475	1	1
at.level(trait, "mic").phylo.342	1	1
at.level(trait, "mic").phylo.471	1	1
at.level(trait, "mic").phylo.264	1	1
at.level(trait, "mic").phylo.517	1	1
at.level(trait, "mic").phylo.507	1	1
at.level(trait, "mic").phylo.132	1	1
at.level(trait, "mic").phylo.39	1	1
at.level(trait, "mic").phylo.286	1	1
at.level(trait, "mic").phylo.524	1	1
at.level(trait, "mic").phylo.533	1	1
at.level(trait, "mic").phylo.333	1	1
at.level(trait, "mic").phylo.410	1	1
at.level(trait, "mic").phylo.446	1	1
at.level(trait, "mic").phylo.344	1	1
at.level(trait, "mic").phylo.406	1	1
at.level(trait, "mic").phylo.22	1	1
at.level(trait, "mic").phylo.430	1	1
at.level(trait, "mic").phylo.288	1	1
at.level(trait, "mic").phylo.511	1	1
at.level(trait, "mic").phylo.429	1	1
at.level(trait, "mic").phylo.277	1	1
at.level(trait, "mic").phylo.322	1	1
at.level(trait, "mic").phylo.262	1	1
at.level(trait, "mic").phylo.387	1	1
at.level(trait, "mic").phylo.58	1	1
at.level(trait, "mic").phylo.388	1	1
at.level(trait, "mic").phylo.158	1	1
at.level(trait, "mic").phylo.12	1	1
at.level(trait, "mic").phylo.276	1	1
at.level(trait, "mic").phylo.412	1	1
at.level(trait, "mic").phylo.411	1	1
at.level(trait, "mic").phylo.365	1	1
at.level(trait, "mic").phylo.82	1	1

at.level(trait, "mic").phylo.92	1	1
at.level(trait, "mic").phylo.370	1	1
at.level(trait, "mic").phylo.137	1	1
at.level(trait, "mic").phylo.267	1	1
at.level(trait, "mic").phylo.421	1	1
at.level(trait, "mic").phylo.237	1	1
at.level(trait, "mic").phylo.200	1	1
at.level(trait, "mic").phylo.536	1	1
at.level(trait, "mic").phylo.260	1	1
at.level(trait, "mic").phylo.537	1	1
at.level(trait, "mic").phylo.477	1	1
at.level(trait, "mic").phylo.518	1	1
at.level(trait, "mic").phylo.55	1	1
at.level(trait, "mic").phylo.358	1	1
at.level(trait, "mic").phylo.197	1	1
at.level(trait, "mic").phylo.493	1	1
at.level(trait, "mic").phylo.41	1	1
at.level(trait, "mic").phylo.40	1	1
at.level(trait, "mic").phylo.408	1	1
at.level(trait, "mic").phylo.292	1	1
at.level(trait, "mic").phylo.248	1	1
at.level(trait, "mic").phylo.1	1	1
at.level(trait, "mic").phylo.214	1	1
at.level(trait, "mic").phylo.480	1	1
at.level(trait, "mic").phylo.423	1	1
at.level(trait, "mic").phylo.247	1	1
at.level(trait, "mic").phylo.268	1	1
at.level(trait, "mic").phylo.353	1	1
at.level(trait, "mic").phylo.211	1	1
at.level(trait, "mic").phylo.265	1	1
at.level(trait, "mic").phylo.139	1	1
at.level(trait, "mic").phylo.198	1	1
at.level(trait, "mic").phylo.396	1	1
at.level(trait, "mic").phylo.263	1	1
at.level(trait, "mic").phylo.95	1	1
at.level(trait, "mic").phylo.162	1	1
at.level(trait, "mic").phylo.515	1	1
at.level(trait, "mic").phylo.282	1	1
at.level(trait, "mic").phylo.427	1	1
at.level(trait, "mic").phylo.240	1	1
at.level(trait, "mic").phylo.166	1	1
at.level(trait, "mic").phylo.182	1	1
at.level(trait, "mic").phylo.532	1	1
at.level(trait, "mic").phylo.273	1	1
at.level(trait, "mic").phylo.119	1	1
at.level(trait, "mic").phylo.355	1	1
at.level(trait, "mic").phylo.241	1	1
at.level(trait, "mic").phylo.203	1	1
at.level(trait, "mic").phylo.121	1	1
at.level(trait, "mic").phylo.305	1	1
at.level(trait, "mic").phylo.534	1	1

at.level(trait, "mic").phylo.167	1	1
at.level(trait, "mic").phylo.336	1	1
at.level(trait, "mic").phylo.188	1	1
at.level(trait, "mic").phylo.274	1	1
at.level(trait, "mic").phylo.281	1	1
at.level(trait, "mic").phylo.89	1	1
at.level(trait, "mic").phylo.531	1	1
at.level(trait, "mic").phylo.164	1	1
at.level(trait, "mic").phylo.382	1	1
at.level(trait, "mic").phylo.192	1	1
at.level(trait, "mic").phylo.301	1	1
at.level(trait, "mic").phylo.506	1	1
at.level(trait, "mic").phylo.334	1	1
at.level(trait, "mic").phylo.469	1	1
at.level(trait, "mic").phylo.2	1	1
at.level(trait, "mic").phylo.470	1	1
at.level(trait, "mic").phylo.351	1	1
at.level(trait, "mic").phylo.31	1	1
at.level(trait, "mic").phylo.159	1	1
at.level(trait, "mic").phylo.187	1	1
at.level(trait, "mic").phylo.488	1	1
at.level(trait, "mic").phylo.486	1	1
at.level(trait, "mic").phylo.225	1	1
at.level(trait, "mic").phylo.32	1	1
at.level(trait, "mic").phylo.541	1	1
at.level(trait, "mic").phylo.380	1	1
at.level(trait, "mic").phylo.462	1	1
at.level(trait, "mic").phylo.451	1	1
at.level(trait, "mic").phylo.403	1	1
at.level(trait, "mic").phylo.115	1	1
at.level(trait, "mic").phylo.250	1	1
at.level(trait, "mic").phylo.93	1	1
at.level(trait, "mic").phylo.230	1	1
at.level(trait, "mic").phylo.457	1	1
at.level(trait, "mic").phylo.313	1	1
at.level(trait, "mic").phylo.331	1	1
at.level(trait, "mic").phylo.176	1	1
at.level(trait, "mic").phylo.75	1	1
at.level(trait, "mic").phylo.296	1	1
at.level(trait, "mic").phylo.205	1	1
at.level(trait, "mic").phylo.256	1	1
at.level(trait, "mic").phylo.487	1	1
at.level(trait, "mic").phylo.347	1	1
at.level(trait, "mic").phylo.442	1	1
at.level(trait, "mic").phylo.495	1	1
at.level(trait, "mic").phylo.116	1	1
at.level(trait, "mic").phylo.233	1	1
at.level(trait, "mic").phylo.539	1	1
at.level(trait, "mic").phylo.83	1	1
at.level(trait, "mic").phylo.444	1	1
at.level(trait, "mic").phylo.508	1	1

at.level(trait, "mic").phylo.33	1	1
at.level(trait, "mic").phylo.142	1	1
at.level(trait, "mic").phylo.311	1	1
at.level(trait, "mic").phylo.386	1	1
at.level(trait, "mic").phylo.135	1	1
at.level(trait, "mic").phylo.19	1	1
at.level(trait, "mic").phylo.59	1	1
at.level(trait, "mic").phylo.287	1	1
at.level(trait, "mic").phylo.375	1	1
at.level(trait, "mic").phylo.490	1	1
at.level(trait, "mic").phylo.448	1	1
at.level(trait, "mic").phylo.10	1	1
at.level(trait, "mic").phylo.399	1	1
at.level(trait, "mic").phylo.466	1	1
at.level(trait, "mic").phylo.310	1	1
at.level(trait, "mic").phylo.543	1	1
at.level(trait, "mic").phylo.504	1	1
at.level(trait, "mic").phylo.174	1	1
at.level(trait, "mic").phylo.499	1	1
at.level(trait, "mic").phylo.126	1	1
at.level(trait, "mic").phylo.413	1	1
at.level(trait, "mic").phylo.125	1	1
at.level(trait, "mic").phylo.201	1	1
at.level(trait, "mic").phylo.513	1	1
at.level(trait, "mic").phylo.69	1	1
at.level(trait, "mic").phylo.340	1	1
at.level(trait, "mic").phylo.179	1	1
at.level(trait, "mic").phylo.25	1	1
at.level(trait, "mic").phylo.436	1	1
at.level(trait, "mic").phylo.546	1	1
at.level(trait, "mic").phylo.279	1	1
at.level(trait, "mic").phylo.485	1	1
at.level(trait, "mic").phylo.38	1	1
at.level(trait, "mic").phylo.422	1	1
at.level(trait, "mic").phylo.229	1	1
at.level(trait, "mic").phylo.35	1	1
at.level(trait, "mic").phylo.131	1	1
at.level(trait, "mic").phylo.71	1	1
at.level(trait, "mic").phylo.6	1	1
at.level(trait, "mic").phylo.401	1	1
at.level(trait, "mic").phylo.222	1	1
at.level(trait, "mic").phylo.243	1	1
at.level(trait, "mic").phylo.42	1	1
at.level(trait, "mic").phylo.63	1	1
at.level(trait, "mic").phylo.18	1	1
at.level(trait, "mic").phylo.123	1	1
at.level(trait, "mic").phylo.140	1	1
at.level(trait, "mic").phylo.502	1	1
at.level(trait, "mic").phylo.503	1	1
at.level(trait, "mic").phylo.341	1	1
at.level(trait, "mic").phylo.238	1	1

```
at.level(trait, "mic").phylo.478                 1          1
at.level(trait, "mic").phylo.443                 1          1
at.level(trait, "mic").phylo.44                  1          1
at.level(trait, "mic").phylo.544                 1          1
at.level(trait, "mic").phylo.204                 1          1
at.level(trait, "mic").phylo.510                 1          1
at.level(trait, "mic").phylo.314                 1          1
at.level(trait, "mic").phylo.124                 1          1
at.level(trait, "mic").phylo.64                  1          1
at.level(trait, "mic").phylo.219                 1          1
at.level(trait, "mic").phylo.178                 1          1
at.level(trait, "mic").phylo.345                 1          1
at.level(trait, "mic").phylo.479                 1          1
at.level(trait, "mic").phylo.307                 1          1
at.level(trait, "mic").phylo.275                 1          1
at.level(trait, "mic").phylo.540                 1          1
at.level(trait, "mic").phylo.23                  1          1
at.level(trait, "mic").phylo.73                  1          1
at.level(trait, "mic").phylo.530                 1          1
at.level(trait, "mic").phylo.244                 1          1
at.level(trait, "mic").phylo.191                 1          1
at.level(trait, "mic").phylo.474                 1          1
at.level(trait, "mic").phylo.460                 1          1
at.level(trait, "mic").phylo.528                 1          1
at.level(trait, "mic").phylo.295                 1          1
at.level(trait, "mic").phylo.459                 1          1
at.level(trait, "mic").phylo.463                 1          1
at.level(trait, "mic").phylo.79                  1          1
at.level(trait, "mic").phylo.481                 1          1
at.level(trait, "mic").phylo.190                 1          1
at.level(trait, "mic").phylo.461                 1          1
at.level(trait, "mic").phylo.535                 1          1
at.level(trait, "mic").phylo.223                 1          1
at.level(trait, "mic").phylo.29                  1          1
at.level(trait, "mic").phylo.65                  1          1
at.level(trait, "mic").phylo.283                 1          1
at.level(trait, "mic").phylo.425                 1          1
at.level(trait, "mic").phylo.107                 1          1
at.level(trait, "mic").phylo.294                 1          1
at.level(trait, "mic").phylo.122                 1          1
at.level(trait, "mic").phylo.105                 1          1
at.level(trait, "mic").phylo.138                 1          1
at.level(trait, "mic").phylo.440                 1          1

Multivariate psrf

1.01
```
