# Supplementary material for: Escherichia coli phylogeny drives co-amoxiclav resistance through variable expression of TEM-1 beta-lactamase
Source: Nat Commun. 2025 Sep 30;16:8669. doi: 10.1038/s41467-025-63714-6 (PMC12484619; doi:10.1038/s41467-025-63714-6)
Supplement: Supplementary file 3 — Description of Additional Supplementary Files [file 41467_2025_63714_MOESM3_ESM.pdf]

Description of Additional Supplementary Files

File Name: Supplementary Data 1
Description: Metadata for all n=377 genomes.

File Name: Supplementary Data 2
Description: Metadata for all n=451 blaTEM-1 annotations

File Name: Supplementary Data 3
Description: qPCR expression data for all replicates.

File Name: Supplementary Data 4
Description: NCBI accessions for short- and long-read sets and assemblies.
