# Supplementary material for: Escherichia coli phylogeny drives co-amoxiclav resistance through variable expression of TEM-1 beta-lactamase
Source: Nat Commun. 2025 Sep 30;16:8669. doi: 10.1038/s41467-025-63714-6 (PMC12484619; doi:10.1038/s41467-025-63714-6)
Supplement: Supplementary file 8 — Reporting Summary [file 41467_2025_63714_MOESM8_ESM.pdf]

nature portfolio

Corresponding author(s): William Matlock (william.matlock@biology.ox.ac.uk); Nicole Stoesser (nicole.stoesser@ndm.ox.ac.uk)

Last updated by author(s): May 20, 2025

Reporting Summary

Nature Portfolio wishes to improve the reproducibility of the work that we publish. This form provides structure for consistency and transparency in reporting. For further information on Nature Portfolio policies, see our Editorial Policies and the Editorial Policy Checklist.

Statistics

For all statistical analyses, confirm that the following items are present in the figure legend, table legend, main text, or Methods section.

n/a	Confirmed	
☐	☒	The exact sample size (*n*) for each experimental group/condition, given as a discrete number and unit of measurement
☐	☒	A statement on whether measurements were taken from distinct samples or whether the same sample was measured repeatedly
☐	☒	The statistical test(s) used AND whether they are one- or two-sided<br>*Only common tests should be described solely by name; describe more complex techniques in the Methods section.*
☐	☒	A description of all covariates tested
☐	☒	A description of any assumptions or corrections, such as tests of normality and adjustment for multiple comparisons
☐	☒	A full description of the statistical parameters including central tendency (e.g. means) or other basic estimates (e.g. regression coefficient) AND variation (e.g. standard deviation) or associated estimates of uncertainty (e.g. confidence intervals)
☐	☒	For null hypothesis testing, the test statistic (e.g. *F*, *t*, *r*) with confidence intervals, effect sizes, degrees of freedom and *P* value noted<br>*Give P values as exact values whenever suitable.*
☐	☒	For Bayesian analysis, information on the choice of priors and Markov chain Monte Carlo settings
☐	☒	For hierarchical and complex designs, identification of the appropriate level for tests and full reporting of outcomes
☒	☐	Estimates of effect sizes (e.g. Cohen's *d*, Pearson's *r*), indicating how they were calculated

Our web collection on statistics for biologists contains articles on many of the points above.

Software and code

Policy information about availability of computer code

Data collection	MinKNOW (v. 21.11.7); Guppy (v. 3.84)
Data analysis	Parameters used are given in "Materials and methods". Software tools and versions: fastp (v. 0.23.4); filtlong (v. 0.2.1); Rasusa (v. 0.7.1); (v. 2.9.2-b1786); bwa (v. 0.7.17-r1188); Polypolish (v. 0.5.0); Unicycler (v. 0.5.0); SPAdes (v. 3.15.5); miniasm (v. 0.3-r179); Racon (v. 1.5.0); Mash screen (v. 2.3); NCBIAMRFinder (v. 3.11.26 and database v. 2023-11- 15.1); tblastn (v. 2.15.0+); Prokka (v . 1.14.6); Abricate (v. 1.0.1); MOB-typer (v. 3.1.4); mlst (v. 2.23.0); EzClermont (v. 0.7.0); MAFFT (v. 7.520); snp-sites (v. 2.5.1); SAMtools (v. 1.18); Panaroo (v. 1.4.2); IQ-Tree (v. 2.3.0); R (v. 4.4.0); RStudio (v. 2024.04.2+764). All scripts used are provided in https://github.com/wtmatlock/tem.

For manuscripts utilizing custom algorithms or software that are central to the research but not yet described in published literature, software must be made available to editors and reviewers. We strongly encourage code deposition in a community repository (e.g. GitHub). See the Nature Portfolio guidelines for submitting code & software for further information.

Data

Policy information about availability of data

All manuscripts must include a data availability statement. This statement should provide the following information, where applicable:

- Accession codes, unique identifiers, or web links for publicly available datasets
- A description of any restrictions on data availability
- For clinical datasets or third party data, please ensure that the statement adheres to our policy

NCBI sequence data accessions (short-/long-reads and assemblies) are provided in Supplementary Table 4. MIC data is provided in Supplementary Table 1. qPCR

data is provided in Supplementary Table 3. Isolate metadata is provided in Supplementary Tables 1 and 2. There are no restrictions on data availability.

Research involving human participants, their data, or biological material

Policy information about studies with human participants or human data. See also policy information about sex, gender (identity/presentation), and sexual orientation and race, ethnicity and racism.

Reporting on sex and gender	NA
Reporting on race, ethnicity, or other socially relevant groupings	NA
Population characteristics	NA
Recruitment	NA
Ethics oversight	The use of genotypic and phenotypic data from these isolates is covered by ethical permissions: London - Queen Square Research Ethics Committee, REC ref 17/LO/1420

Note that full information on the approval of the study protocol must also be provided in the manuscript.

Field-specific reporting

Please select the one below that is the best fit for your research. If you are not sure, read the appropriate sections before making your selection.

☐ Life sciences ☐ Behavioural & social sciences ☒ Ecological, evolutionary & environmental sciences

For a reference copy of the document with all sections, see nature.com/documents/nr-reporting-summary-flat.pdf

Ecological, evolutionary & environmental sciences study design

All studies must disclose on these points even when the disclosure is negative.

Study description	A genotype (377 E. coli hybrid assemblies) to phenotype (co-amoxiclav MIC and blaTEM-1 expression) modelling study. We employ Bayesian modelling techniques that control for phylogeny. Genotype features are extracted with bioinformatics methods.
Research sample	E. coli isolates cultured from human bloodstream infections (BSI) from patients presenting to Oxford University Hospitals NHS Foundation Trust.
Sampling strategy	All isolates were collected as part of a larger genomic surveillance study of systematically sequenced isolates from patients presenting to Oxford University Hospitals NHS Foundation Trust 2008-2018: https://www.ebi.ac.uk/ena/browser/view/PRJNA604975
Data collection	Of the original surveillance study: all isolates caused BSI between September 15, 2008, and December 01, 2018 (de-duplicated to one BSI isolate per 90-day period per patient), and were processed by the clinical microbiology laboratory at the John Radcliffe Hospital, Oxford, UK.
Timing and spatial scale	Isolates used range from 2013-2018, and all from hospital patients in Oxfordshire, UK.
Data exclusions	n=548 candidate isolates were filtered down to n=377 based on pre-determined exclusion criteria as follows: after genome assembly, we removed n=171/548 isolates, either because (i) the chromosome did not circularise (116/171), (ii) it carried a non-blaTEM-1 blaTEM variant and/or an additional acquired beta-lactamase (54/171), or (iii) the chromosome was too short consistent with misassembly (~3.5Mbp; 1/171). This left a final dataset of n=377 isolates.
Reproducibility	For blaTEM-1 qPCR expression data, biological and technical replicates were made as described in Materials and methods. All scripts used are provided in https://github.com/wtmatlock/tem and stably archived at https://doi.org/10.5281/zenodo.15829498.
Randomization	NA
Blinding	NA

Did the study involve field work? ☐ Yes ☒ No

Reporting for specific materials, systems and methods

We require information from authors about some types of materials, experimental systems and methods used in many studies. Here, indicate whether each material, system or method listed is relevant to your study. If you are not sure if a list item applies to your research, read the appropriate section before selecting a response.

Materials & experimental systems

n/a	Involved in the study
☒	☐ Antibodies
☒	☐ Eukaryotic cell lines
☒	☐ Palaeontology and archaeology
☒	☐ Animals and other organisms
☒	☐ Clinical data
☒	☐ Dual use research of concern
☒	☐ Plants

Methods

n/a	Involved in the study
☒	☐ ChIP-seq
☒	☐ Flow cytometry
☒	☐ MRI-based neuroimaging

Plants

Seed stocks	NA
Novel plant genotypes	NA
Authentication	NA
